# Comparative proteomics in captive giant pandas to identify proteins involved in age-related cataract formation

**DOI:** 10.1038/s41598-023-40003-0

**Published:** 2023-08-05

**Authors:** Yuyan You, Chao Bai, Wei Wang, Tongtong Zhan, Xin Hu, Feier Hao, Maohua Xia, Yan Liu, Tao Ma, Yanhui Liu, Changming Zheng, Tianchun Pu, Yizhuo Zhang, Yanping Lu, Nan Ding, Jing Li, Yanqiang Yin, Yucun Chen, Liqin Wang, Jun Zhou, Lili Niu, Yunfang Xiu, Yan Lu, Ting Jia, Xuefeng Liu, Chenglin Zhang

**Affiliations:** 1 Beijing Key Laboratory of Captive Wildlife Technologies, Beijing Zoo, Beijing, China; 2 Beijing Zoo, Beijing, China; 3 Chongqing Zoo, Chongqing, China; 4 Strait (Fuzhou) Giant Panda Research and Exchange Centers, Fuzhou, China; 5 Chengdu Zoo, Chengdu, China

**Keywords:** Zoology, Diseases

## Abstract

Approximately 20% of aged captive giant pandas (*Ailuropoda melanoleuca*) have cataracts that impair their quality of life. To identify potential biomarkers of cataract formation, we carried out a quantitative proteomics analysis of 10 giant pandas to find proteins differing in abundance between healthy and cataract-bearing animals. We identified almost 150 proteins exceeding our threshold for differential abundance, most of which were associated with GO categories related to extracellular localization. The most significant differential abundance was associated with components of the proteasome and other proteins with a role in proteolysis or its regulation, most of which were depleted in pandas with cataracts. Other modulated proteins included components of the extracellular matrix or cytoskeleton, as well as associated signaling proteins and regulators, but we did not find any differentially expressed transcription factors. These results indicate that the formation of cataracts involves a complex post-transcriptional network of signaling inside and outside lens cells to drive stress responses as a means to address the accumulation of protein aggregates triggered by oxidative damage. The modulated proteins also indicate that it should be possible to predict the onset of cataracts in captive pandas by taking blood samples and testing them for the presence or absence of specific protein markers.

## Introduction

Animals in captivity live longer than those in the wild because they are largely protected from predators, internecine competition, and resource scarcity. However, this exposes such animals to diseases of aging that are not encountered at a high frequency in wild populations, including age-related cataracts^1^. Such cataracts can be heritable, but with significant environmental triggers, including oxidative stress in the lens and the resulting accumulation of DNA and protein damage^2–4^. This is the most common cause of blindness in humans and long-lived companion animals^5–7^ and it now has a prevalence of ~ 20% in the aging captive population of giant pandas (*Ailuropoda melanoleuca*)^8^, which live 10–15 years longer in captivity compared to those in the wild^9,10^.

Genetic analysis has revealed numerous genes associated with cataracts, encoding proteins related to oxidative stress responses, the production of antioxidant enzymes and metabolites, and various DNA repair pathways^11–13^. Previous studies in several mammalian species have investigated the regulation of transcription during the development of cataracts as well as differences in DNA methylation and/or RNA profiles between normal and affected individuals^14–16^. In our previous work, we identified 110 candidate genes that are differentially methylated in giant pandas with and without cataracts (including six genes associated with age-related cataracts in humans)^17^ as well as more than 700 differentially expressed transcripts^18^. However, the vast majority of gene products that act as effectors during cataract formation are proteins rather than RNA molecules, and changes at the RNA level do not always correlate directly with changes at the protein level. The proteomic analysis of pandas with and without cataracts may thus provide greater insight into the molecular basis of age-related cataracts in the giant panda population.

We therefore took blood samples from four aged giant pandas with cataracts and six healthy controls for quantitative proteomics analysis using tandem mass tags. We identified proteins that were exclusively or primarily present in healthy pandas or those with cataracts, and assigned them to functional classes and pathways. The analysis of the panda cataract proteome will identify key proteins involved in cataract formation and the resulting pathology, providing new information that could lead to the development of preventative strategies, new diagnostic assays, and non-invasive therapies to reduce the disease burden in the aging panda population.

## Materials and methods

### Ethical aspects

All samples were collected in accordance with the Wildlife Protection Law of the People’s Republic of China (President of the People’s Republic of China No. 16), and the sampling procedure and subsequent experiments were approved by the Beijing Zoo Academic and Ethics Committee. All methods are reported in accordance with ARRIVE guidelines (https://arriveguidelines.org).

### Clinical description of test subjects

Peripheral blood samples (2 ml) were collected from 10 giant pandas (seven females and three males) ranging in age from 21 to 39 (Table 1). Giant pandas 18 or years or older are defined as aged because the equivalent human age is ~ 75. Four of the females (three deceased) were diagnosed with age-related cataracts and the other three females and all three males were healthy, cataract-free controls. We carefully reviewed the case files of each subject to rule out other diseases and traumatic sources of cataract before we selected them for analysis. Importantly, the Beijing Zoo Academic and Ethics Committee insisted that we restrict the number of aged pandas used in the experiments because they are particularly vulnerable. Therefore, the control group was not matched to the age of the cataract group, but instead represented a range of ages and thus helped to rule-out general age-related (rather than specific cataract-related) effects. Blood samples were used because they are easier to obtain than eye biopsies. However, were able to obtain one lens from a deceased panda with cataracts and we analyzed the anterior capsule. The transcriptome data were consistent with the corresponding blood sample (data not shown), which suggested that blood testing is an adequate surrogate for the direct testing of eye material. This approach is much more convenient for the predictive testing of pandas in the future. In the single living female panda with cataracts, the lens capsule had shrunk, the anterior chamber had deepened, the nucleus of the lens had subsided, the cortical granules of the lens had accumulated in the anterior chamber angle and blocked the trabecular meshwork, and we observed lens capsular degeneration and cortical spillage, lens opacification, white coloration, and increased nuclear hardness, similar to human nuclear hardness grade VI. However, this grading was only noted for reference and does not represent the actual hardness of the panda lens. This is because there is no direct correspondence between the cataract disease stages in humans and giant pandas.

**Table 1:** Description of giant panda sample donors.

Sample	Sex	Birth year	Status	Remarks
A1	Female	1986	Cataracts	Died
A2	Female	1993	Cataracts	Died
A3	Female	1982	Cataracts	Died
A4	Female	1986	Cataracts	Died
B1	Female	1999	Healthy	Alive
B2	Female	1990	Healthy	Alive
B3	Female	2013	Healthy	Alive
C1	Male	2013	Healthy	Alive
C2	Male	1999	Healthy	Alive
C3	Male	2000	Healthy	Alive

Sample band A features the affected females, whereas bands B and C feature the healthy females and males, respectively.

### Protein extraction

The blood samples were centrifuged to prepare serum, which was aliquoted and stored at – 80 °C. For proteomic analysis, 40-µL aliquots of serum were diluted in binding buffer and loaded onto a Pierce Albumin/IgG Removal Kit column (Thermo Fisher Scientific, Waltham, MA, USA). After two rounds of treatment according to the manufacturer’s instructions, the depleted serum was eluted, lyophilized, and stored at – 80 °C. The lyophilized samples were redissolved in 300 µL SDS lysis buffer and centrifuged at 12,000×*g* for 15 min at 5 °C to remove insoluble particles. After repeating this step, the protein concentration was determined using a bicinchoninic acid (BCA) kit (Shengong Biological Technology, Shanghai, China) and aliquots of each sample were used for SDS-PAGE and quantitative proteomics.

### SDS-PAGE

Reconstituted depleted serum aliquots containing 10 µg of total protein were separated by SDS-PAGE on 12% polyacrylamide gels. The gels were post-stained with Coomassie Brilliant Blue (Sigma-Aldrich, St Louis, MO, USA) and the stained gels were documented using an ImageScanner (GE Healthcare, Chicago, IL, USA).

### Tryptic digestion and mass tag labeling

Reconstituted depleted serum aliquots containing 10 µg of total protein were digested with trypsin according to the filter-aided sample preparation (FASP) protocol^19^ with some modifications. Briefly, each protein sample was reduced with 10 mM dithiothreitol, 8 M urea and 100 mM triethylammonium bicarbonate (TEAB) buffer (pH 8.0) for 1 h at 60 °C, then alkylated with 50 mM iodoacetamide at room temperature for 40 min before centrifugal filtration at 12,000×*g* (10-kDa cut-off) for 20 min at room temperature. The filtrate was diluted in 100 µL 300 mM TEAB buffer and the centrifugation step was repeated twice. The filtrate was transferred to a fresh tube containing 100 µL 300 mM TEAB buffer, and on-filter digestion was performed overnight at 37 °C using 0.1 μg/μL sequencing-grade trypsin (Promega, Madison, WI, USA). The peptides were eluted by centrifugation at 12,000×*g* for 20 min at room temperature with one change of TEAB buffer, and the final eluate was lyophilized.

For mass tag labeling, the lyophilized peptides were reconstituted in 100 μL 200 mM TEAB buffer and stored at room temperature. The tandem mass tag (TMT) reagent (Thermo Fisher Scientific) was mixed with acetonitrile and centrifuged according to the manufacturer’s instructions. This reagent consists of three chemical groups (reporter, balance and reaction groups) that have the same combined mass (producing a single peak in MS1) but split into different masses during fragmentation (producing separate peaks in MS2 that can be quantified across multiple samples). The peptides and TMT reagent were mixed and allowed to react at room temperature for 1 h before we terminated the reaction with 8 μL 5% hydroxylamine for 15 min. The labeled peptides were lyophilized and stored at – 80 °C.

### RP-HPLC–MS

Samples were separated on a Zorbax Extend C18 narrow-diameter column (2.1 × 150 mm, 5 μm; Agilent Technologies, Santa Clara, CA, USA) mounted on an Agilent 1100 HPLC system at a flow rate of 300 μL/min in a gradient of mobile phases A (water containing 2% v/v acetonitrile) and B (90% v/v acetonitrile in water). The gradient elution profile was: 0–8 min, 98% A; 8–8.01 min, 98–95% A; 8.01–38 min, 95–75% A; 38–50 min, 75–60% A; 50–50.01 min, 60–10% A; 50.01–60 min, 10% A; 60–60.01 min, 10–98% A, 60.01–65 min, 98% A. The samples were freeze-dried under vacuum and stored for MS analysis.

The samples were fractionated at a flow rate of 300 nL/min on an Acclaim PepMap RSLC C18 column (75 μm × 150 mm, 2 μm, 100 Å; Thermo Fisher Scientific) mounted on an EASY-nLCTM 1200 liquid phase system (Thermo Fisher Scientific) and were eluted in a gradient of mobile phases A (0.1% formic acid in water) and B (80% v/v acetonitrile in water with 0.1% formic acid). The gradient elution profile was: 0–55 min, 8% B; 55–79 min, 30% B; 79–80 min, 50% B; 80–90 min, 100% B. The separated peptides were introduced into a Thermo Q-active mass spectrometer (Thermo Fisher Scientific) via a nano-liter spray ion source (Thermo Fisher Scientific). The mass resolution of MS1 was set to 70,000, the automatic gain control value was 1 × 10^6^, and the scanning range was 300–1600 m*/z*. The 10 most intense peaks were scanned in MS^2^ by high-energy collisional fragmentation in data-dependent positive ion mode, with the collision energy set to 32 eV, the resolution set to 17,500, the automatic gain control set to 2 × 10^5^, the maximum ion accumulation time set to 80 ms, and the dynamic exclusion time set to 15 s.

### Proteomic data analysis

Proteomic data were analyzed using Xcalibur v2.1 and were screened against the UniProt panda database using Proteome Discoverer v2.2 (Thermo Fisher Scientific). The false discovery rate (FDR) for peptide identification was held below 1%. The screening criteria to classify differentially expressed proteins were a fold change of at least 0.5 and a significance of p < 0.05. The resulting datasets are available in the ProteomeXchange Consortium repository (http://proteomecentral.proteomexchange.org/cgi/GetDataset?ID=PXD031039 or https://www.iprox.cn/page/project.html?id=IPX0004000000; Project ID IPX0004000000, accession number PXD031039). Quantitative bias within samples caused by differences in amino acid sequence length was eliminated by applying the IBAQ method as previously described^20^. Furthermore, quantitative bias across samples caused by sampling and/or loading errors was eliminated by using the total quantity method, which consisted of (i) adding the IBAQ values of each protein identified in the sample to represent the total amount of protein in the sample, (ii) dividing the IBAQ of each protein in the sample by the total amount of protein in the sample, and (iii) multiplying this value by 100,000 to obtain the normalized protein quantitative value. Variation between the cataract and control groups was compared to within-group variation by one-way ANOVA (SPSS Statistics; IBM, Armonk, NY, USA).

### Functional analysis of proteins differing in abundance between cataract and control samples

The TMT quantitative proteomics data were screened for proteins differing in abundance between the pandas with cataracts (samples A1–A4) and healthy controls (samples in bands B or C or B + C). All differentially expressed proteins were then used as search queries against the Gene Ontology and KEGG pathways database, as well as databases of protein interactions, allowing the analysis of comparative expression profiles as well as the construction of heat maps, Venn diagrams, and protein interaction network maps^21–23^. Functional enrichment analysis was carried out to identify differentially expressed proteins significantly enriched in GO terms (biological processes, cellular localization and molecular functions) or KEGG metabolic pathways. Genes were mapped to the GO and KEGG databases, the number of proteins representing each term or pathway was calculated, and hypergeometric tests were applied to identify significantly enriched GO terms or KEGG pathways in the protein list. GO terms and KEGG pathways were considered significant at q < 0.05.

## Results

### Protein sample preparation and assessment for quality and quantity

Blood samples from 10 giant panda specimens were used to prepare depleted serum proteins for further analysis. To confirm the quality and quantity of protein in the depleted samples, which were prepared by passing serum through an albumin/IgG capture column, we measured the protein concentration using a BCA assay followed by absorbance spectrophotometry, and separated the samples by SDS-PAGE to determine the integrity of the major bands. The protein concentrations in each sample are shown in Table 2.

**Table 2:** Triplicate absorbance readings (and average reading) and the calculated concentration of proteins in the analyte (diluted for measurement) and the original depleted serum sample.

Sample	Absorbance 1	Absorbance 2	Absorbance 3	Average absorbance	Analyte concentration (μg/μL)	Sample concentration (μg/μL)
A1	0.428	0.431	0.444	0.434	0.424	4.244
A2	0.375	0.378	0.395	0.383	0.374	3.740
A3	0.349	0.353	0.36	0.354	0.346	3.461
A4	0.417	0.415	0.432	0.421	0.412	4.117
B1	0.3	0.307	0.315	0.307	0.301	3.005
B2	0.321	0.326	0.339	0.329	0.321	3.213
B3	0.319	0.317	0.333	0.323	0.316	3.158
C1	0.321	0.316	0.326	0.321	0.314	3.139
C2	0.317	0.316	0.328	0.320	0.313	3.132
C3	0.335	0.328	0.337	0.333	0.326	3.259

### Protein identification and overview of differential expression

**Figure 1:**
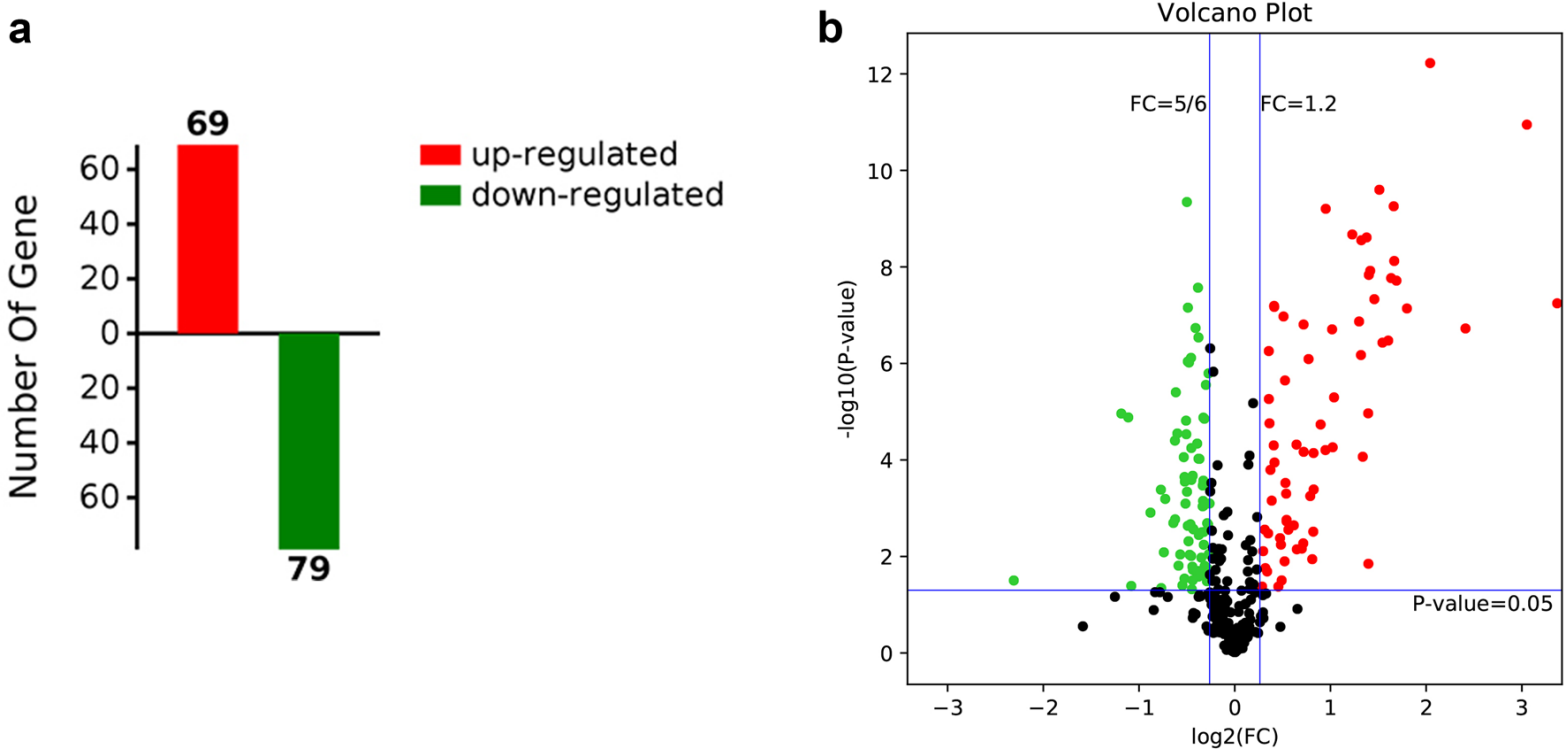
Differential protein expression when comparing band A (pandas with cataracts) to bands B and C combined (pandas without cataracts). (**a**) The number of upregulated and downregulated proteins in band A compared to bands B + C. (**b**) Volcano plot showing the most meaningful differentially expressed proteins in band A compared to bands B + C by plotting significance on the y-axis against fold-change on the x-axis. Proteins to the left (green dots) are downregulated and those to the right (red dots) are upregulated according to the statistical threshold, below which the proteins are not considered to be differentially expressed (black dots).

LC–MS/MS analysis and database searching resulted in the identification of 342 proteins with qualitative data and 318 with quantitative data meeting the criterion FDR < 1%. We focused on the differences between the pandas in band A (with cataracts) and those in bands B and C combined (healthy controls) in order to maximize the likelihood of recovering differentially expressed proteins reflecting the presence or absence of cataracts rather than those reflecting other differences, such as female vs male. This comparison revealed a total of 148 modulated proteins, 69 upregulated and 79 downregulated in the pandas with cataracts (Fig. 1a). This was based on a fold-change in abundance of ≥ 0.5 and a significance of p < 0.05, as represented in the corresponding volcano plot (Fig. 1b). Among these differentially expressed proteins, 25 of the upregulated proteins and four of the downregulated proteins were characterized by a log_2_ fold-change > 1 (Table 3 and Supplementary Table S1). There were also 75 upregulated and 96 downregulated proteins in the comparison A vs B (Supplementary Fig. S1) and 81 upregulated and 81 downregulated proteins in the comparison A vs C (Supplementary Fig. S2). A heat map showing the clustering of the proteins in each sample is provided in Supplementary Figure S3. The variation between the cataract and control groups was significantly greater than the variation within either group (one-way ANOVA, F = 7.888, p = 0.000401).

**Table 3:** Partial list of differentially expressed proteins (cut-off at |log_2_ fold change| > 1).

No.	ID	Uniprot	Genename	Log2FoldChange	Biological process	Cell component	Molecular function	KEGG pathways	UNIPROT_URL	Transcription factor
1	G1LNL2	G1LNL2	ENSAMEG00000008217	1.660934345		GO:0044425, membrane part|GO:0016021, integral component of membrane|GO:0016020, membrane|GO:0031224, intrinsic component of membrane			http://www.uniprot.org/uniprot/G1LNL2	FALSE
2	G1LPA5	G1LPA5	ENSAMEG00000008427	1.458651392					http://www.uniprot.org/uniprot/G1LPA5	FALSE
3	G1LUD1	G1LUD1	ENSAMEG00000010166	1.227872351					http://www.uniprot.org/uniprot/G1LUD1	FALSE
4	G1MB17	G1MB17	APCS	− 1.112413519	GO:0080090, regulation of primary metabolic process|GO:0019222, regulation of metabolic process|GO:0030224, monocyte differentiation|GO:0052203, modulation of catalytic activity in other organism involved in symbiotic interaction|GO:0051701, interaction with host|GO:0052205, modulation of molecular function in other organism involved in symbiotic interaction|GO:0052204, negative regulation of molecular function in other organism involved in symbiotic interaction|GO:0010605, negative regulation of macromolecule metabolic process|GO:0071704, organic substance metabolic process|GO:0048869, cellular developmental process|GO:0044419, interspecies interaction between organisms|GO:0048513, animal organ development|GO:0051817, modification of morphology or physiology of other organism involved in symbiotic interaction|GO:0044092, negative regulation of molecular function|GO:0044793, negative regulation by host of viral process|GO:1903016, negative regulation of exo-alpha-sialidase activity|GO:1903015, regulation of exo-alpha-sialidase activity|GO:0052405, negative regulation by host of symbiont molecular function|GO:1903018, regulation of glycoprotein metabolic process|GO:1903019, negative regulation of glycoprotein metabolic process|GO:0044868, modulation by host of viral molecular function|GO:0044869, negative regulation by host of viral exo-alpha-sialidase activity|GO:0051702, interaction with symbiont|GO:0044866, modulation by host of viral exo-alpha-sialidase activity|GO:0044867, modulation by host of viral catalytic activity|GO:1903706, regulation of hemopoiesis|GO:1903707, negative regulation of hemopoiesis|GO:0051704, multi-organism process|GO:0044359, modulation of molecular function in other organism|GO:0044707, single-multicellular organism process|GO:0019538, protein metabolic process|GO:0045655, regulation of monocyte differentiation|GO:0045656, negative regulation of monocyte differentiation|GO:0002376, immune system process|GO:0044788, modulation by host of viral process|GO:0046718, viral entry into host cell|GO:0009892, negative regulation of metabolic process|GO:0044871, negative regulation by host of viral glycoprotein metabolic process|GO:0044870, modulation by host of viral glycoprotein metabolic process|GO:0050789, regulation of biological process|GO:0002761, regulation of myeloid leukocyte differentiation|GO:0002762, negative regulation of myeloid leukocyte differentiation|GO:0051346, negative regulation of hydrolase activity|GO:0044260, cellular macromolecule metabolic process|GO:0002682, regulation of immune system process|GO:0002683, negative regulation of immune system process|GO:0065009, regulation of molecular function|GO:0065008, regulation of biological quality|GO:0052422, modulation by host of symbiont catalytic activity|GO:0050793, regulation of developmental process|GO:0050792, regulation of viral process|GO:0050790, regulation of catalytic activity|GO:0052428, modification by host of symbiont molecular function|GO:0006952, defense response|GO:0043903, regulation of symbiosis, encompassing mutualism through parasitism|GO:0043900, regulation of multi-organism process|GO:0043901, negative regulation of multi-organism process|GO:0051239, regulation of multicellular organismal process|GO:0030097, hemopoiesis|GO:0050794, regulation of cellular process|GO:0002521, leukocyte differentiation|GO:0002520, immune system development|GO:0051336, regulation of hydrolase activity|GO:0050896, response to stimulus|GO:0006950, response to stress|GO:0045638, negative regulation of myeloid cell differentiation|GO:0045637, regulation of myeloid cell differentiation|GO:0008152, metabolic process|GO:0030154, cell differentiation|GO:0030260, entry into host cell|GO:0052126, movement in host environment|GO:0043086, negative regulation of catalytic activity|GO:0044699, single-organism process|GO:0044362, negative regulation of molecular function in other organism|GO:0051248, negative regulation of protein metabolic process|GO:0052055, modulation by symbiont of host molecular function|GO:1903900, regulation of viral life cycle|GO:1903901, negative regulation of viral life cycle|GO:0051241, negative regulation of multicellular organismal process|GO:0051246, regulation of protein metabolic process|GO:0051851, modification by host of symbiont morphology or physiology|GO:0019058, viral life cycle|GO:0032502, developmental process|GO:0032501, multicellular organismal process|GO:0009987, cellular process|GO:0045596, negative regulation of cell differentiation|GO:0045595, regulation of cell differentiation|GO:0006955, immune response|GO:0048519, negative regulation of biological process|GO:0051093, negative regulation of developmental process|GO:1901135, carbohydrate derivative metabolic process|GO:0043170, macromolecule metabolic process|GO:0051828, entry into other organism involved in symbiotic interaction|GO:0030099, myeloid cell differentiation|GO:0048731, system development|GO:0048856, anatomical structure development|GO:0031324, negative regulation of cellular metabolic process|GO:0031323, regulation of cellular metabolic process|GO:0046596, regulation of viral entry into host cell|GO:0046597, negative regulation of viral entry into host cell|GO:0009100, glycoprotein metabolic process|GO:0007275, multicellular organism development|GO:0002573, myeloid leukocyte differentiation|GO:0060255, regulation of macromolecule metabolic process|GO:0048534, hematopoietic or lymphoid organ development|GO:0045087, innate immune response|GO:0044767, single-organism developmental process|GO:0044764, multi-organism cellular process|GO:0044763, single-organism cellular process|GO:0052403, negative regulation by host of symbiont catalytic activity|GO:0044003, modification by symbiont of host morphology or physiology|GO:0040011, locomotion|GO:0044238, primary metabolic process|GO:0040013, negative regulation of locomotion|GO:0040012, regulation of locomotion|GO:0052199, negative regulation of catalytic activity in other organism involved in symbiotic interaction|GO:0065007, biological regulation|GO:0052192, movement in environment of other organism involved in symbiotic interaction|GO:0044237, cellular metabolic process|GO:1902106, negative regulation of leukocyte differentiation|GO:1902105, regulation of leukocyte differentiation|GO:0044409, entry into host|GO:2000026, regulation of multicellular organismal development|GO:0016032, viral process|GO:0048525, negative regulation of viral process|GO:0044403, symbiosis, encompassing mutualism through parasitism|GO:0051806, entry into cell of other organism involved in symbiotic interaction|GO:0035821, modification of morphology or physiology of other organism|GO:0048523, negative regulation of cellular process|GO:1901564, organonitrogen compound metabolic process|GO:1903131, mononuclear cell differentiation|GO:0006807, nitrogen compound metabolic process	GO:0043231, intracellular membrane-bounded organelle|GO:0005622, intracellular|GO:0043230, extracellular organelle|GO:0070062, extracellular exosome|GO:0044421, extracellular region part|GO:0043227, membrane-bounded organelle|GO:0005634, nucleus|GO:0044464, cell part|GO:0005623, cell|GO:0031988, membrane-bounded vesicle|GO:0031012, extracellular matrix|GO:0043229, intracellular organelle|GO:0005576, extracellular region|GO:0005615, extracellular space|GO:0072562, blood microparticle|GO:0044424, intracellular part|GO:1903561, extracellular vesicle|GO:0065010, extracellular membrane-bounded organelle|GO:0043226, organelle|GO:0031982, vesicle	GO:0005488, binding|GO:0043169, cation binding|GO:0001849, complement component C1q binding|GO:0043167, ion binding|GO:0046790, virion binding|GO:0001848, complement binding|GO:0005509, calcium ion binding|GO:0005515, protein binding|GO:0046872, metal ion binding|GO:0001846, opsonin binding		http://www.uniprot.org/uniprot/G1MB17	FALSE
5	G1MJI0	G1MJI0	FGA	1.400881793	GO:2001234, negative regulation of apoptotic signaling pathway|GO:0034116, positive regulation of heterotypic cell-cell adhesion|GO:2001236, regulation of extrinsic apoptotic signaling pathway|GO:2001237, negative regulation of extrinsic apoptotic signaling pathway|GO:0019220, regulation of phosphate metabolic process|GO:0080090, regulation of primary metabolic process|GO:0019222, regulation of metabolic process|GO:2001233, regulation of apoptotic signaling pathway|GO:0051049, regulation of transport|GO:0048585, negative regulation of response to stimulus|GO:0048584, positive regulation of response to stimulus|GO:0048583, regulation of response to stimulus|GO:0019229, regulation of vasoconstriction|GO:0007160, cell-matrix adhesion|GO:0045907, positive regulation of vasoconstriction|GO:0007165, signal transduction|GO:0007166, cell surface receptor signaling pathway|GO:0003018, vascular process in circulatory system|GO:0034113, heterotypic cell-cell adhesion|GO:0023014, signal transduction by protein phosphorylation|GO:0044710, single-organism metabolic process|GO:0010604, positive regulation of macromolecule metabolic process|GO:0009968, negative regulation of signal transduction|GO:0045785, positive regulation of cell adhesion|GO:0009966, regulation of signal transduction|GO:0009967, positive regulation of signal transduction|GO:0051047, positive regulation of secretion|GO:0000165, MAPK cascade|GO:0009611, response to wounding|GO:0046879, hormone secretion|GO:0019731, antibacterial humoral response|GO:0048518, positive regulation of biological process|GO:0048519, negative regulation of biological process|GO:0033036, macromolecule localization|GO:0007599, hemostasis|GO:0031589, cell-substrate adhesion|GO:0007596, blood coagulation|GO:0051050, positive regulation of transport|GO:0060255, regulation of macromolecule metabolic process|GO:0060548, negative regulation of cell death|GO:0009914, hormone transport|GO:0045184, establishment of protein localization|GO:0090276, regulation of peptide hormone secretion|GO:0090277, positive regulation of peptide hormone secretion|GO:0007155, cell adhesion|GO:0010038, response to metal ion|GO:0009607, response to biotic stimulus|GO:0010035, response to inorganic substance|GO:0051707, response to other organism|GO:0030168, platelet activation|GO:0003008, system process|GO:0042325, regulation of phosphorylation|GO:0044700, single organism signaling|GO:0042327, positive regulation of phosphorylation|GO:0065008, regulation of biological quality|GO:0051716, cellular response to stimulus|GO:0016192, vesicle-mediated transport|GO:0044707, single-multicellular organism process|GO:0019538, protein metabolic process|GO:0002376, immune system process|GO:0003013, circulatory system process|GO:0050880, regulation of blood vessel size|GO:0098609, cell-cell adhesion|GO:1903530, regulation of secretion by cell|GO:0035150, regulation of tube size|GO:0022607, cellular component assembly|GO:0009893, positive regulation of metabolic process|GO:0006950, response to stress|GO:1903532, positive regulation of secretion by cell|GO:0008104, protein localization|GO:0032940, secretion by cell|GO:0065007, biological regulation|GO:0042742, defense response to bacterium|GO:0042310, vasoconstriction|GO:0035556, intracellular signal transduction|GO:0051223, regulation of protein transport|GO:0042981, regulation of apoptotic process|GO:0050789, regulation of biological process|GO:0030072, peptide hormone secretion|GO:0009605, response to external stimulus|GO:0044267, cellular protein metabolic process|GO:0050708, regulation of protein secretion|GO:0044260, cellular macromolecule metabolic process|GO:0006887, exocytosis|GO:0016043, cellular component organization|GO:0090066, regulation of anatomical structure size|GO:0065003, macromolecular complex assembly|GO:0008015, blood circulation|GO:1904035, regulation of epithelial cell apoptotic process|GO:0071840, cellular component organization or biogenesis|GO:1904036, negative regulation of epithelial cell apoptotic process|GO:0032880, regulation of protein localization|GO:0016265, obsolete death|GO:0009617, response to bacterium|GO:0070201, regulation of establishment of protein localization|GO:0098602, single organism cell adhesion|GO:0009306, protein secretion|GO:0006810, transport|GO:0042886, amide transport|GO:0042060, wound healing|GO:0050794, regulation of cellular process|GO:0043410, positive regulation of MAPK cascade|GO:0012501, programmed cell death|GO:1903524, positive regulation of blood circulation|GO:0050817, coagulation|GO:1903522, regulation of blood circulation|GO:0006464, cellular protein modification process|GO:0051239, regulation of multicellular organismal process|GO:0006955, immune response|GO:1902533, positive regulation of intracellular signal transduction|GO:1902531, regulation of intracellular signal transduction|GO:0006952, defense response|GO:0046903, secretion|GO:0072577, endothelial cell apoptotic process|GO:0043207, response to external biotic stimulus|GO:0050714, positive regulation of protein secretion|GO:0043412, macromolecule modification|GO:0050896, response to stimulus|GO:0031401, positive regulation of protein modification process|GO:0001775, cell activation|GO:0034114, regulation of heterotypic cell-cell adhesion|GO:0036211, protein modification process|GO:0043152, induction of bacterial agglutination|GO:0051046, regulation of secretion|GO:0051240, positive regulation of multicellular organismal process|GO:1902042, negative regulation of extrinsic apoptotic signaling pathway via death domain receptors|GO:1902041, regulation of extrinsic apoptotic signaling pathway via death domain receptors|GO:0006461, protein complex assembly|GO:0008152, metabolic process|GO:0070271, protein complex biogenesis|GO:0016310, phosphorylation|GO:0030155, regulation of cell adhesion|GO:0023056, positive regulation of signaling|GO:0023057, negative regulation of signaling|GO:0015833, peptide transport|GO:0023052, signaling|GO:0010648, negative regulation of cell communication|GO:0023051, regulation of signaling|GO:0051592, response to calcium ion|GO:0010647, positive regulation of cell communication|GO:0010646, regulation of cell communication|GO:0044699, single-organism process|GO:0043408, regulation of MAPK cascade|GO:1904019, epithelial cell apoptotic process|GO:0008625, extrinsic apoptotic signaling pathway via death domain receptors|GO:0044057, regulation of system process|GO:0010562, positive regulation of phosphorus metabolic process|GO:0051246, regulation of protein metabolic process|GO:0051247, positive regulation of protein metabolic process|GO:0032270, positive regulation of cellular protein metabolic process|GO:0031399, regulation of protein modification process|GO:0022610, biological adhesion|GO:0017157, regulation of exocytosis|GO:0032501, multicellular organismal process|GO:0050878, regulation of body fluid levels|GO:0031323, regulation of cellular metabolic process|GO:0009987, cellular process|GO:2000351, regulation of endothelial cell apoptotic process|GO:2000352, negative regulation of endothelial cell apoptotic process|GO:0060627, regulation of vesicle-mediated transport|GO:0046883, regulation of hormone secretion|GO:0098542, defense response to other organism|GO:0046887, positive regulation of hormone secretion|GO:0032879, regulation of localization|GO:0051258, protein polymerization|GO:0032268, regulation of cellular protein metabolic process|GO:0090087, regulation of peptide transport|GO:0043170, macromolecule metabolic process|GO:0070374, positive regulation of ERK1 and ERK2 cascade|GO:0070372, regulation of ERK1 and ERK2 cascade|GO:0070371, ERK1 and ERK2 cascade|GO:0045921, positive regulation of exocytosis|GO:0016337, single organismal cell-cell adhesion|GO:0060341, regulation of cellular localization|GO:0043933, macromolecular complex subunit organization|GO:0031325, positive regulation of cellular metabolic process|GO:0097190, apoptotic signaling pathway|GO:0097191, extrinsic apoptotic signaling pathway|GO:0022407, regulation of cell-cell adhesion|GO:0006959, humoral immune response|GO:0034622, cellular macromolecular complex assembly|GO:0051234, establishment of localization|GO:0008219, cell death|GO:0010941, regulation of cell death|GO:0051222, positive regulation of protein transport|GO:0022409, positive regulation of cell-cell adhesion|GO:0042221, response to chemical|GO:0072377, blood coagulation, common pathway|GO:0072376, protein activation cascade|GO:0072378, blood coagulation, fibrin clot formation|GO:0071705, nitrogen compound transport|GO:0071704, organic substance metabolic process|GO:0043067, regulation of programmed cell death|GO:0043066, negative regulation of apoptotic process|GO:0071702, organic substance transport|GO:0051704, multi-organism process|GO:0043069, negative regulation of programmed cell death|GO:0097285, obsolete cell-type specific apoptotic process|GO:0006468, protein phosphorylation|GO:0045937, positive regulation of phosphate metabolic process|GO:0034109, homotypic cell-cell adhesion|GO:0006915, apoptotic process|GO:0023061, signal release|GO:0010817, regulation of hormone levels|GO:0051174, regulation of phosphorus metabolic process|GO:0044765, single-organism transport|GO:0044763, single-organism cellular process|GO:0051649, establishment of localization in cell|GO:0007267, cell-cell signaling|GO:0007154, cell communication|GO:0019730, antimicrobial humoral response|GO:0043623, cellular protein complex assembly|GO:0051179, localization|GO:1902578, single-organism localization|GO:0051641, cellular localization|GO:0044238, primary metabolic process|GO:0071822, protein complex subunit organization|GO:0002790, peptide secretion|GO:0002791, regulation of peptide secretion|GO:0002793, positive regulation of peptide secretion|GO:0044237, cellular metabolic process|GO:0070527, platelet aggregation|GO:0006796, phosphate-containing compound metabolic process|GO:0044085, cellular component biogenesis|GO:0006793, phosphorus metabolic process|GO:0015031, protein transport|GO:0001932, regulation of protein phosphorylation|GO:0001934, positive regulation of protein phosphorylation|GO:0048523, negative regulation of cellular process|GO:0048522, positive regulation of cellular process|GO:0010467, gene expression|GO:1901564, organonitrogen compound metabolic process|GO:0030193, regulation of blood coagulation|GO:0030195, negative regulation of blood coagulation|GO:1900046, regulation of hemostasis|GO:1900047, negative regulation of hemostasis|GO:0050818, regulation of coagulation|GO:0050819, negative regulation of coagulation|GO:0042730, fibrinolysis|GO:0051604, protein maturation|GO:0080134, regulation of response to stress|GO:0032102, negative regulation of response to external stimulus|GO:0032101, regulation of response to external stimulus|GO:1904951, positive regulation of establishment of protein localization|GO:0051241, negative regulation of multicellular organismal process|GO:0006508, proteolysis|GO:1903034, regulation of response to wounding|GO:1903035, negative regulation of response to wounding|GO:0016485, protein processing|GO:0006807, nitrogen compound metabolic process|GO:0061041, regulation of wound healing|GO:0061045, negative regulation of wound healing|GO:0031639, plasminogen activation|GO:0031638, zymogen activation	GO:0009897, external side of plasma membrane|GO:0043229, intracellular organelle|GO:0071944, cell periphery|GO:0098552, side of membrane|GO:0043227, membrane-bounded organelle|GO:0043226, organelle|GO:0005737, cytoplasm|GO:0031091, platelet alpha granule|GO:0070062, extracellular exosome|GO:0016023, cytoplasmic, membrane-bounded vesicle|GO:0031410, cytoplasmic vesicle|GO:0016020, membrane|GO:0044444, cytoplasmic part|GO:0072562, blood microparticle|GO:0031988, membrane-bounded vesicle|GO:0005615, extracellular space|GO:0044459, plasma membrane part|GO:0030141, secretory granule|GO:0009986, cell surface|GO:0005938, cell cortex|GO:0005886, plasma membrane|GO:0032991, macromolecular complex|GO:1903561, extracellular vesicle|GO:0031982, vesicle|GO:0043234, protein complex|GO:0043230, extracellular organelle|GO:0043231, intracellular membrane-bounded organelle|GO:0044464, cell part|GO:0005623, cell|GO:0005622, intracellular|GO:0005577, fibrinogen complex|GO:0005576, extracellular region|GO:0044424, intracellular part|GO:0044425, membrane part|GO:0065010, extracellular membrane-bounded organelle|GO:0044421, extracellular region part|GO:0012505, endomembrane system|GO:0099503, secretory vesicle|GO:0099568, cytoplasmic region|GO:0097708, intracellular vesicle	GO:0030674, protein binding, bridging|GO:0005198, structural molecule activity|GO:0005488, binding|GO:0005515, protein binding|GO:0005102, receptor binding|GO:0050839, cell adhesion molecule binding|GO:0060090, binding, bridging		http://www.uniprot.org/uniprot/G1MJI0	FALSE
6	G1LKP1	G1LKP1	ENSAMEG00000007135	1.377829229					http://www.uniprot.org/uniprot/G1LKP1	FALSE
7	G1MMG4	G1MMG4	ENSAMEG00000019512	1.299197134					http://www.uniprot.org/uniprot/G1MMG4	FALSE
8	G1M4J5	G1M4J5	ENSAMEG00000013547	1.037606126					http://www.uniprot.org/uniprot/G1M4J5	FALSE
9	Q7M3C1	Q7M3C1	MB	1.322543678	GO:0006810, transport|GO:0015669, gas transport|GO:0044765, single-organism transport|GO:0015671, oxygen transport|GO:1902578, single-organism localization|GO:0051234, establishment of localization|GO:0051179, localization|GO:0044699, single-organism process		GO:0043169, cation binding|GO:0005344, oxygen transporter activity|GO:0022892, substrate-specific transporter activity|GO:0046914, transition metal ion binding|GO:0020037, heme binding|GO:0046906, tetrapyrrole binding|GO:0005215, transporter activity|GO:0043167, ion binding|GO:0019825, oxygen binding|GO:0005488, binding|GO:0097159, organic cyclic compound binding|GO:0005506, iron ion binding|GO:0046872, metal ion binding|GO:1901363, heterocyclic compound binding		http://www.uniprot.org/uniprot/Q7M3C1	FALSE
10	G1KZU2	G1KZU2	POSTN	− 1.081168123	GO:0048583, regulation of response to stimulus|GO:0023052, signaling|GO:0007165, signal transduction|GO:0007166, cell surface receptor signaling pathway|GO:0023051, regulation of signaling|GO:0010646, regulation of cell communication|GO:0050789, regulation of biological process|GO:0071840, cellular component organization or biogenesis|GO:0051716, cellular response to stimulus|GO:0007219, Notch signaling pathway|GO:0009966, regulation of signal transduction|GO:0016043, cellular component organization|GO:0065007, biological regulation|GO:0044699, single-organism process|GO:0043062, extracellular structure organization|GO:0022610, biological adhesion|GO:0032502, developmental process|GO:0009987, cellular process|GO:0009888, tissue development|GO:0050794, regulation of cellular process|GO:0044763, single-organism cellular process|GO:0008593, regulation of Notch signaling pathway|GO:0007155, cell adhesion|GO:0007154, cell communication|GO:0044700, single organism signaling|GO:0050896, response to stimulus|GO:0048856, anatomical structure development|GO:0030198, extracellular matrix organization|GO:0009991, response to extracellular stimulus|GO:0070887, cellular response to chemical stimulus|GO:0071295, cellular response to vitamin|GO:0031670, cellular response to nutrient|GO:1901655, cellular response to ketone|GO:0033273, response to vitamin|GO:0071496, cellular response to external stimulus|GO:0032571, response to vitamin K|GO:1901701, cellular response to oxygen-containing compound|GO:0007584, response to nutrient|GO:0042221, response to chemical|GO:0010033, response to organic substance|GO:1901700, response to oxygen-containing compound|GO:0031668, cellular response to extracellular stimulus|GO:0031669, cellular response to nutrient levels|GO:0009605, response to external stimulus|GO:0031667, response to nutrient levels|GO:1901654, response to ketone|GO:0071307, cellular response to vitamin K	GO:0031012, extracellular matrix|GO:0043229, intracellular organelle|GO:0043227, membrane-bounded organelle|GO:0043226, organelle|GO:0005737, cytoplasm|GO:0031984, organelle subcompartment|GO:0044431, Golgi apparatus part|GO:0005794, Golgi apparatus|GO:0012505, endomembrane system|GO:0043231, intracellular membrane-bounded organelle|GO:0005802, trans-Golgi network|GO:0044464, cell part|GO:0005623, cell|GO:0005622, intracellular|GO:0044446, intracellular organelle part|GO:0044444, cytoplasmic part|GO:0005576, extracellular region|GO:0098791, Golgi subcompartment|GO:0005578, proteinaceous extracellular matrix|GO:0044424, intracellular part|GO:0044421, extracellular region part|GO:0044422, organelle part|GO:0005615, extracellular space	GO:1901681, sulfur compound binding|GO:0043168, anion binding|GO:0097367, carbohydrate derivative binding|GO:0043167, ion binding|GO:0005539, glycosaminoglycan binding|GO:0005488, binding|GO:0008201, heparin binding|GO:0043169, cation binding|GO:0046872, metal ion binding		http://www.uniprot.org/uniprot/G1KZU2	FALSE
11	D2H7X8	D2H7X8	FTL	1.799120339	GO:0050801, ion homeostasis|GO:0042592, homeostatic process|GO:0055072, iron ion homeostasis|GO:0006826, iron ion transport|GO:0055076, transition metal ion homeostasis|GO:0098771, inorganic ion homeostasis|GO:0044699, single-organism process|GO:0046916, cellular transition metal ion homeostasis|GO:0000041, transition metal ion transport|GO:0065007, biological regulation|GO:0065008, regulation of biological quality|GO:0019725, cellular homeostasis|GO:0006810, transport|GO:0006879, cellular iron ion homeostasis|GO:0006875, cellular metal ion homeostasis|GO:0006812, cation transport|GO:0006811, ion transport|GO:0009987, cellular process|GO:0006873, cellular ion homeostasis|GO:0044765, single-organism transport|GO:0044763, single-organism cellular process|GO:0030003, cellular cation homeostasis|GO:0055065, metal ion homeostasis|GO:0055080, cation homeostasis|GO:0055082, cellular chemical homeostasis|GO:0051234, establishment of localization|GO:0051179, localization|GO:1902578, single-organism localization|GO:0048878, chemical homeostasis|GO:0030001, metal ion transport	GO:0005623, cell	GO:0043169, cation binding|GO:0046914, transition metal ion binding|GO:0043167, ion binding|GO:0008199, ferric iron binding|GO:0005488, binding|GO:0005506, iron ion binding|GO:0046872, metal ion binding	aml04978, Mineral absorption	http://www.uniprot.org/uniprot/D2H7X8	FALSE
12	G1MKA5	G1MKA5	ENSAMEG00000018739	1.666462743					http://www.uniprot.org/uniprot/G1MKA5	FALSE
13	G1LQ06	G1LQ06	ENSAMEG00000008683	1.017268912					http://www.uniprot.org/uniprot/G1LQ06	FALSE
14	G1MIQ3	G1MIQ3	SAAL1	3.369050286	GO:0050896, response to stimulus|GO:0006952, defense response|GO:0006953, acute-phase response|GO:0006950, response to stress|GO:0006954, inflammatory response|GO:0002526, acute inflammatory response	GO:0005576, extracellular region			http://www.uniprot.org/uniprot/G1MIQ3	FALSE
15	G1MJI1	G1MJI1	FGG	1.510917875	GO:2001234, negative regulation of apoptotic signaling pathway|GO:0034116, positive regulation of heterotypic cell-cell adhesion|GO:2001236, regulation of extrinsic apoptotic signaling pathway|GO:2001237, negative regulation of extrinsic apoptotic signaling pathway|GO:0019220, regulation of phosphate metabolic process|GO:0080090, regulation of primary metabolic process|GO:0019222, regulation of metabolic process|GO:2001233, regulation of apoptotic signaling pathway|GO:0051049, regulation of transport|GO:0048585, negative regulation of response to stimulus|GO:0048584, positive regulation of response to stimulus|GO:0048583, regulation of response to stimulus|GO:0019229, regulation of vasoconstriction|GO:0007160, cell-matrix adhesion|GO:0045907, positive regulation of vasoconstriction|GO:0007165, signal transduction|GO:0007166, cell surface receptor signaling pathway|GO:0003018, vascular process in circulatory system|GO:0034113, heterotypic cell-cell adhesion|GO:0023014, signal transduction by protein phosphorylation|GO:0044710, single-organism metabolic process|GO:0010604, positive regulation of macromolecule metabolic process|GO:0009968, negative regulation of signal transduction|GO:0045785, positive regulation of cell adhesion|GO:0009966, regulation of signal transduction|GO:0009967, positive regulation of signal transduction|GO:0051047, positive regulation of secretion|GO:0000165, MAPK cascade|GO:0009611, response to wounding|GO:0046879, hormone secretion|GO:0048518, positive regulation of biological process|GO:0048519, negative regulation of biological process|GO:0033036, macromolecule localization|GO:0007599, hemostasis|GO:0031589, cell-substrate adhesion|GO:0007596, blood coagulation|GO:0051050, positive regulation of transport|GO:0060255, regulation of macromolecule metabolic process|GO:0060548, negative regulation of cell death|GO:0009914, hormone transport|GO:0045184, establishment of protein localization|GO:0090276, regulation of peptide hormone secretion|GO:0090277, positive regulation of peptide hormone secretion|GO:0007155, cell adhesion|GO:0010038, response to metal ion|GO:0010035, response to inorganic substance|GO:0030168, platelet activation|GO:0003008, system process|GO:0042325, regulation of phosphorylation|GO:0044700, single organism signaling|GO:0042327, positive regulation of phosphorylation|GO:0065008, regulation of biological quality|GO:0051716, cellular response to stimulus|GO:0016192, vesicle-mediated transport|GO:0044707, single-multicellular organism process|GO:0019538, protein metabolic process|GO:0003013, circulatory system process|GO:0050880, regulation of blood vessel size|GO:0098609, cell-cell adhesion|GO:1903530, regulation of secretion by cell|GO:0035150, regulation of tube size|GO:0022607, cellular component assembly|GO:0009893, positive regulation of metabolic process|GO:0006950, response to stress|GO:1903532, positive regulation of secretion by cell|GO:0008104, protein localization|GO:0032940, secretion by cell|GO:0065007, biological regulation|GO:0042310, vasoconstriction|GO:0035556, intracellular signal transduction|GO:0051223, regulation of protein transport|GO:0042981, regulation of apoptotic process|GO:0050789, regulation of biological process|GO:0030072, peptide hormone secretion|GO:0044267, cellular protein metabolic process|GO:0050708, regulation of protein secretion|GO:0044260, cellular macromolecule metabolic process|GO:0006887, exocytosis|GO:0016043, cellular component organization|GO:0090066, regulation of anatomical structure size|GO:0065003, macromolecular complex assembly|GO:0008015, blood circulation|GO:1904035, regulation of epithelial cell apoptotic process|GO:0071840, cellular component organization or biogenesis|GO:1904036, negative regulation of epithelial cell apoptotic process|GO:0032880, regulation of protein localization|GO:0016265, obsolete death|GO:0070201, regulation of establishment of protein localization|GO:0098602, single organism cell adhesion|GO:0009306, protein secretion|GO:0006810, transport|GO:0042886, amide transport|GO:0042060, wound healing|GO:0050794, regulation of cellular process|GO:0043410, positive regulation of MAPK cascade|GO:0012501, programmed cell death|GO:1903524, positive regulation of blood circulation|GO:0050817, coagulation|GO:1903522, regulation of blood circulation|GO:0006464, cellular protein modification process|GO:0051239, regulation of multicellular organismal process|GO:1902533, positive regulation of intracellular signal transduction|GO:1902531, regulation of intracellular signal transduction|GO:0046903, secretion|GO:0072577, endothelial cell apoptotic process|GO:0050714, positive regulation of protein secretion|GO:0043412, macromolecule modification|GO:0050896, response to stimulus|GO:0031401, positive regulation of protein modification process|GO:0001775, cell activation|GO:0034114, regulation of heterotypic cell-cell adhesion|GO:0036211, protein modification process|GO:0051046, regulation of secretion|GO:0051240, positive regulation of multicellular organismal process|GO:1902042, negative regulation of extrinsic apoptotic signaling pathway via death domain receptors|GO:1902041, regulation of extrinsic apoptotic signaling pathway via death domain receptors|GO:0006461, protein complex assembly|GO:0008152, metabolic process|GO:0070271, protein complex biogenesis|GO:0016310, phosphorylation|GO:0030155, regulation of cell adhesion|GO:0023056, positive regulation of signaling|GO:0023057, negative regulation of signaling|GO:0015833, peptide transport|GO:0023052, signaling|GO:0010648, negative regulation of cell communication|GO:0023051, regulation of signaling|GO:0051592, response to calcium ion|GO:0010647, positive regulation of cell communication|GO:0010646, regulation of cell communication|GO:0044699, single-organism process|GO:0043408, regulation of MAPK cascade|GO:1904019, epithelial cell apoptotic process|GO:0008625, extrinsic apoptotic signaling pathway via death domain receptors|GO:0044057, regulation of system process|GO:0010562, positive regulation of phosphorus metabolic process|GO:0051246, regulation of protein metabolic process|GO:0051247, positive regulation of protein metabolic process|GO:0032270, positive regulation of cellular protein metabolic process|GO:0031399, regulation of protein modification process|GO:0022610, biological adhesion|GO:0017157, regulation of exocytosis|GO:0032501, multicellular organismal process|GO:0050878, regulation of body fluid levels|GO:0031323, regulation of cellular metabolic process|GO:0009987, cellular process|GO:2000351, regulation of endothelial cell apoptotic process|GO:2000352, negative regulation of endothelial cell apoptotic process|GO:0060627, regulation of vesicle-mediated transport|GO:0046883, regulation of hormone secretion|GO:0046887, positive regulation of hormone secretion|GO:0032879, regulation of localization|GO:0051258, protein polymerization|GO:0032268, regulation of cellular protein metabolic process|GO:0090087, regulation of peptide transport|GO:0043170, macromolecule metabolic process|GO:0070374, positive regulation of ERK1 and ERK2 cascade|GO:0070372, regulation of ERK1 and ERK2 cascade|GO:0070371, ERK1 and ERK2 cascade|GO:0045921, positive regulation of exocytosis|GO:0016337, single organismal cell-cell adhesion|GO:0060341, regulation of cellular localization|GO:0043933, macromolecular complex subunit organization|GO:0031325, positive regulation of cellular metabolic process|GO:0097190, apoptotic signaling pathway|GO:0097191, extrinsic apoptotic signaling pathway|GO:0022407, regulation of cell-cell adhesion|GO:0034622, cellular macromolecular complex assembly|GO:0051234, establishment of localization|GO:0008219, cell death|GO:0010941, regulation of cell death|GO:0051222, positive regulation of protein transport|GO:0022409, positive regulation of cell-cell adhesion|GO:0042221, response to chemical|GO:0071822, protein complex subunit organization|GO:0071705, nitrogen compound transport|GO:0071704, organic substance metabolic process|GO:0043067, regulation of programmed cell death|GO:0043066, negative regulation of apoptotic process|GO:0071702, organic substance transport|GO:0043069, negative regulation of programmed cell death|GO:0097285, obsolete cell-type specific apoptotic process|GO:0006468, protein phosphorylation|GO:0045937, positive regulation of phosphate metabolic process|GO:0034109, homotypic cell-cell adhesion|GO:0006915, apoptotic process|GO:0023061, signal release|GO:0010817, regulation of hormone levels|GO:0051174, regulation of phosphorus metabolic process|GO:0044765, single-organism transport|GO:0044763, single-organism cellular process|GO:0051649, establishment of localization in cell|GO:0007267, cell-cell signaling|GO:0007154, cell communication|GO:0043623, cellular protein complex assembly|GO:0051179, localization|GO:1902578, single-organism localization|GO:0051641, cellular localization|GO:0044238, primary metabolic process|GO:0002790, peptide secretion|GO:0002791, regulation of peptide secretion|GO:0002793, positive regulation of peptide secretion|GO:0044237, cellular metabolic process|GO:0070527, platelet aggregation|GO:0006796, phosphate-containing compound metabolic process|GO:0044085, cellular component biogenesis|GO:0006793, phosphorus metabolic process|GO:0015031, protein transport|GO:0001932, regulation of protein phosphorylation|GO:0001934, positive regulation of protein phosphorylation|GO:0048523, negative regulation of cellular process|GO:0048522, positive regulation of cellular process|GO:0010467, gene expression|GO:1901564, organonitrogen compound metabolic process|GO:0030193, regulation of blood coagulation|GO:0030195, negative regulation of blood coagulation|GO:1900046, regulation of hemostasis|GO:1900047, negative regulation of hemostasis|GO:0050818, regulation of coagulation|GO:0050819, negative regulation of coagulation|GO:0042730, fibrinolysis|GO:0051604, protein maturation|GO:0080134, regulation of response to stress|GO:0032102, negative regulation of response to external stimulus|GO:0032101, regulation of response to external stimulus|GO:1904951, positive regulation of establishment of protein localization|GO:0051241, negative regulation of multicellular organismal process|GO:0006508, proteolysis|GO:1903034, regulation of response to wounding|GO:1903035, negative regulation of response to wounding|GO:0016485, protein processing|GO:0006807, nitrogen compound metabolic process|GO:0061041, regulation of wound healing|GO:0061045, negative regulation of wound healing|GO:0072376, protein activation cascade|GO:0072378, blood coagulation, fibrin clot formation|GO:0031639, plasminogen activation|GO:0031638, zymogen activation|GO:0009605, response to external stimulus	GO:0009897, external side of plasma membrane|GO:0043229, intracellular organelle|GO:0071944, cell periphery|GO:0098552, side of membrane|GO:0043227, membrane-bounded organelle|GO:0043226, organelle|GO:0005737, cytoplasm|GO:0031091, platelet alpha granule|GO:0070062, extracellular exosome|GO:0016023, cytoplasmic, membrane-bounded vesicle|GO:0031410, cytoplasmic vesicle|GO:0016020, membrane|GO:0044444, cytoplasmic part|GO:0072562, blood microparticle|GO:0031988, membrane-bounded vesicle|GO:0005615, extracellular space|GO:0044459, plasma membrane part|GO:0030141, secretory granule|GO:0009986, cell surface|GO:0005938, cell cortex|GO:0005886, plasma membrane|GO:0032991, macromolecular complex|GO:1903561, extracellular vesicle|GO:0031982, vesicle|GO:0043234, protein complex|GO:0043230, extracellular organelle|GO:0043231, intracellular membrane-bounded organelle|GO:0044464, cell part|GO:0005623, cell|GO:0005622, intracellular|GO:0005577, fibrinogen complex|GO:0005576, extracellular region|GO:0044424, intracellular part|GO:0044425, membrane part|GO:0065010, extracellular membrane-bounded organelle|GO:0044421, extracellular region part|GO:0012505, endomembrane system|GO:0099503, secretory vesicle|GO:0097708, intracellular vesicle	GO:0030674, protein binding, bridging|GO:0005198, structural molecule activity|GO:0005488, binding|GO:0005515, protein binding|GO:0005102, receptor binding|GO:0050839, cell adhesion molecule binding|GO:0060090, binding, bridging		http://www.uniprot.org/uniprot/G1MJI1	FALSE
16	D2GUY3	D2GUY3	TPM4	− 1.185094746	GO:0032502, developmental process|GO:0032501, multicellular organismal process|GO:0044707, single-multicellular organism process|GO:0048869, cellular developmental process|GO:0030154, cell differentiation|GO:0044767, single-organism developmental process|GO:0001503, ossification|GO:0044763, single-organism cellular process|GO:0044699, single-organism process|GO:0009987, cellular process|GO:0001649, osteoblast differentiation	GO:0032432, actin filament bundle|GO:0015629, actin cytoskeleton|GO:0043229, intracellular organelle|GO:0043228, non-membrane-bounded organelle|GO:0005924, cell-substrate adherens junction|GO:0005925, focal adhesion|GO:0043227, membrane-bounded organelle|GO:0043226, organelle|GO:0030054, cell junction|GO:0042641, actomyosin|GO:0005856, cytoskeleton|GO:0001725, stress fiber|GO:0005938, cell cortex|GO:0002102, podosome|GO:0031982, vesicle|GO:0016020, membrane|GO:0031988, membrane-bounded vesicle|GO:0044430, cytoskeletal part|GO:0030055, cell-substrate junction|GO:0005576, extracellular region|GO:0005737, cytoplasm|GO:0031941, filamentous actin|GO:0070161, anchoring junction|GO:0005884, actin filament|GO:0032991, macromolecular complex|GO:1903561, extracellular vesicle|GO:0070062, extracellular exosome|GO:0043234, protein complex|GO:0043230, extracellular organelle|GO:0043232, intracellular non-membrane-bounded organelle|GO:0030863, cortical cytoskeleton|GO:0044464, cell part|GO:0005623, cell|GO:0005622, intracellular|GO:0044446, intracellular organelle part|GO:0044444, cytoplasmic part|GO:0071944, cell periphery|GO:0044448, cell cortex part|GO:0044424, intracellular part|GO:0065010, extracellular membrane-bounded organelle|GO:0044421, extracellular region part|GO:0044422, organelle part|GO:0005912, adherens junction|GO:0099512, supramolecular fiber|GO:0097517, contractile actin filament bundle|GO:0099568, cytoplasmic region|GO:0099513, polymeric cytoskeletal fiber		aml05410, Hypertrophic cardiomyopathy (HCM)|aml05414, Dilated cardiomyopathy|aml04260, Cardiac muscle contraction|aml04261, Adrenergic signaling in cardiomyocytes	http://www.uniprot.org/uniprot/D2GUY3	FALSE
17	G1L780	G1L780	ENSAMEG00000002626	1.543822928					http://www.uniprot.org/uniprot/G1L780	FALSE
18	G1KZS5	G1KZS5	LOC100483473	1.337494957					http://www.uniprot.org/uniprot/G1KZS5	FALSE
19	G1LSF9	G1LSF9	ENSAMEG00000009525	1.415438304					http://www.uniprot.org/uniprot/G1LSF9	FALSE
20	G1MJV9	G1MJV9	ENSAMEG00000018591	1.394244417					http://www.uniprot.org/uniprot/G1MJV9	FALSE
21	G1LKE7	G1LKE7	ENSAMEG00000007039	1.603611757					http://www.uniprot.org/uniprot/G1LKE7	FALSE
22	G1M4V6	G1M4V6	FGL1	1.318851165		GO:0043230, extracellular organelle|GO:0031982, vesicle|GO:0044421, extracellular region part|GO:0043227, membrane-bounded organelle|GO:0031988, membrane-bounded vesicle|GO:0005576, extracellular region|GO:1903561, extracellular vesicle|GO:0065010, extracellular membrane-bounded organelle|GO:0043226, organelle|GO:0070062, extracellular exosome			http://www.uniprot.org/uniprot/G1M4V6	FALSE
23	G1M8B3	G1M8B3	IGFBP1	2.41111805	GO:0016049, cell growth|GO:0040007, growth|GO:0040008, regulation of growth|GO:0009987, cellular process|GO:0016043, cellular component organization|GO:0050794, regulation of cellular process|GO:0001558, regulation of cell growth|GO:0044763, single-organism cellular process|GO:0071840, cellular component organization or biogenesis|GO:0065007, biological regulation|GO:0051128, regulation of cellular component organization|GO:0050789, regulation of biological process|GO:0044699, single-organism process	GO:0005576, extracellular region|GO:0005615, extracellular space|GO:0044421, extracellular region part|GO:0005737, cytoplasm|GO:0005794, Golgi apparatus|GO:0043231, intracellular membrane-bounded organelle|GO:0044464, cell part|GO:0044444, cytoplasmic part|GO:0005623, cell|GO:0005622, intracellular|GO:0043229, intracellular organelle|GO:0012505, endomembrane system|GO:0044424, intracellular part|GO:0043227, membrane-bounded organelle|GO:0043226, organelle	GO:0005520, insulin-like growth factor binding|GO:0019838, growth factor binding|GO:0005515, protein binding|GO:0005488, binding|GO:0031994, insulin-like growth factor I binding|GO:0031995, insulin-like growth factor II binding		http://www.uniprot.org/uniprot/G1M8B3	FALSE
24	G1L3J9	G1L3J9	CAMP	1.689489419	GO:0006952, defense response|GO:0006950, response to stress|GO:0050896, response to stimulus|GO:0045926, negative regulation of growth|GO:0044139, modulation of growth of symbiont on or near host|GO:0044110, growth involved in symbiotic interaction|GO:0042742, defense response to bacterium|GO:0044116, growth of symbiont involved in interaction with host|GO:0044117, growth of symbiont in host|GO:0050829, defense response to Gram-negative bacterium|GO:0050789, regulation of biological process|GO:0044130, negative regulation of growth of symbiont in host|GO:0044140, negative regulation of growth of symbiont on or near host surface|GO:0040007, growth|GO:0043207, response to external biotic stimulus|GO:0044364, disruption of cells of other organism|GO:0040008, regulation of growth|GO:0009617, response to bacterium|GO:0051852, disruption by host of symbiont cells|GO:0051851, modification by host of symbiont morphology or physiology|GO:0044133, growth of symbiont on or near host|GO:0044419, interspecies interaction between organisms|GO:0065007, biological regulation|GO:0051873, killing by host of symbiont cells|GO:0048519, negative regulation of biological process|GO:0001906, cell killing|GO:0051818, disruption of cells of other organism involved in symbiotic interaction|GO:0051704, multi-organism process|GO:0045087, innate immune response|GO:0043903, regulation of symbiosis, encompassing mutualism through parasitism|GO:0043900, regulation of multi-organism process|GO:0043901, negative regulation of multi-organism process|GO:0098542, defense response to other organism|GO:0006955, immune response|GO:0051702, interaction with symbiont|GO:0044126, regulation of growth of symbiont in host|GO:0051707, response to other organism|GO:0044146, negative regulation of growth of symbiont involved in interaction with host|GO:0044144, modulation of growth of symbiont involved in interaction with host|GO:0009607, response to biotic stimulus|GO:0009605, response to external stimulus|GO:0051883, killing of cells in other organism involved in symbiotic interaction|GO:0031640, killing of cells of other organism|GO:0050830, defense response to Gram-positive bacterium|GO:0002376, immune system process|GO:0044403, symbiosis, encompassing mutualism through parasitism|GO:0035821, modification of morphology or physiology of other organism|GO:0051817, modification of morphology or physiology of other organism involved in symbiotic interaction	GO:0005576, extracellular region|GO:0005737, cytoplasm|GO:0012505, endomembrane system|GO:0043231, intracellular membrane-bounded organelle|GO:0099503, secretory vesicle|GO:0016023, cytoplasmic, membrane-bounded vesicle|GO:0030141, secretory granule|GO:0044464, cell part|GO:0044444, cytoplasmic part|GO:0005623, cell|GO:0031988, membrane-bounded vesicle|GO:0042581, specific granule|GO:0043229, intracellular organelle|GO:0097708, intracellular vesicle|GO:0031410, cytoplasmic vesicle|GO:0044424, intracellular part|GO:0005622, intracellular|GO:0043227, membrane-bounded organelle|GO:0043226, organelle|GO:0031982, vesicle			http://www.uniprot.org/uniprot/G1L3J9	FALSE
25	G1M307	G1M307	RGN	1.0231726	GO:0019222, regulation of metabolic process|GO:0050790, regulation of catalytic activity|GO:0065007, biological regulation|GO:0008152, metabolic process|GO:0065009, regulation of molecular function|GO:0050789, regulation of biological process		GO:0043169, cation binding|GO:0098772, molecular function regulator|GO:0030234, enzyme regulator activity|GO:0043167, ion binding|GO:0005509, calcium ion binding|GO:0046872, metal ion binding|GO:0005488, binding	aml00053, Ascorbate and aldarate metabolism|aml01200, Carbon metabolism|aml01100, Metabolic pathways|aml00030, Pentose phosphate pathway	http://www.uniprot.org/uniprot/G1M307	FALSE
26	G1LGI1	G1LGI1	BHMT	2.041739123	GO:0044272, sulfur compound biosynthetic process|GO:0071267, L-methionine salvage|GO:0019752, carboxylic acid metabolic process|GO:0071265, L-methionine biosynthetic process|GO:0044249, cellular biosynthetic process|GO:0034641, cellular nitrogen compound metabolic process|GO:0006807, nitrogen compound metabolic process|GO:0009067, aspartate family amino acid biosynthetic process|GO:0009066, aspartate family amino acid metabolic process|GO:0044283, small molecule biosynthetic process|GO:0044699, single-organism process|GO:1901576, organic substance biosynthetic process|GO:0044710, single-organism metabolic process|GO:0044711, single-organism biosynthetic process|GO:0006520, cellular amino acid metabolic process|GO:0071704, organic substance metabolic process|GO:0043102, amino acid salvage|GO:0032259, methylation|GO:1901605, alpha-amino acid metabolic process|GO:1901607, alpha-amino acid biosynthetic process|GO:0009987, cellular process|GO:0044238, primary metabolic process|GO:0000097, sulfur amino acid biosynthetic process|GO:0000096, sulfur amino acid metabolic process|GO:0009058, biosynthetic process|GO:0044763, single-organism cellular process|GO:0008152, metabolic process|GO:0043436, oxoacid metabolic process|GO:0043094, cellular metabolic compound salvage|GO:0008652, cellular amino acid biosynthetic process|GO:1901564, organonitrogen compound metabolic process|GO:1901566, organonitrogen compound biosynthetic process|GO:0006082, organic acid metabolic process|GO:0006577, amino-acid betaine metabolic process|GO:0046394, carboxylic acid biosynthetic process|GO:0006555, methionine metabolic process|GO:0016053, organic acid biosynthetic process|GO:0044237, cellular metabolic process|GO:0006790, sulfur compound metabolic process|GO:0009086, methionine biosynthetic process|GO:0044281, small molecule metabolic process	GO:0005737, cytoplasm|GO:0005622, intracellular|GO:0043230, extracellular organelle|GO:0031982, vesicle|GO:0044421, extracellular region part|GO:0043227, membrane-bounded organelle|GO:0044464, cell part|GO:0005623, cell|GO:0031988, membrane-bounded vesicle|GO:0005576, extracellular region|GO:0044424, intracellular part|GO:1903561, extracellular vesicle|GO:0065010, extracellular membrane-bounded organelle|GO:0043226, organelle|GO:0070062, extracellular exosome|GO:0005829, cytosol|GO:0044444, cytoplasmic part	GO:0043169, cation binding|GO:0008898, S-adenosylmethionine-homocysteine S-methyltransferase activity|GO:0046914, transition metal ion binding|GO:0008757, S-adenosylmethionine-dependent methyltransferase activity|GO:0043167, ion binding|GO:0008172, S-methyltransferase activity|GO:0003824, catalytic activity|GO:0016740, transferase activity|GO:0016741, transferase activity, transferring one-carbon groups|GO:0008270, zinc ion binding|GO:0047150, betaine-homocysteine S-methyltransferase activity|GO:0008168, methyltransferase activity|GO:0046872, metal ion binding|GO:0005488, binding	aml00260, Glycine, serine and threonine metabolism|aml01100, Metabolic pathways|aml00270, Cysteine and methionine metabolism	http://www.uniprot.org/uniprot/G1LGI1	FALSE
27	G1LUE9	G1LUE9	IGKV4-1	1.634152524		GO:0005576, extracellular region|GO:0005615, extracellular space|GO:0072562, blood microparticle|GO:0044421, extracellular region part|GO:0043230, extracellular organelle|GO:0070062, extracellular exosome|GO:1903561, extracellular vesicle|GO:0043227, membrane-bounded organelle|GO:0043226, organelle|GO:0031982, vesicle			http://www.uniprot.org/uniprot/G1LUE9	FALSE
28	G1LXP2	G1LXP2	WHSC1L1	1.396286325	GO:0006479, protein methylation|GO:0080090, regulation of primary metabolic process|GO:0019222, regulation of metabolic process|GO:0051568, histone H3-K4 methylation|GO:1901362, organic cyclic compound biosynthetic process|GO:1901360, organic cyclic compound metabolic process|GO:0044710, single-organism metabolic process|GO:0010605, negative regulation of macromolecule metabolic process|GO:0018193, peptidyl-amino acid modification|GO:0016571, histone methylation|GO:0016570, histone modification|GO:0060255, regulation of macromolecule metabolic process|GO:2001141, regulation of RNA biosynthetic process|GO:0046483, heterocycle metabolic process|GO:0070734, histone H3-K27 methylation|GO:0019538, protein metabolic process|GO:0018205, peptidyl-lysine modification|GO:0019438, aromatic compound biosynthetic process|GO:0016568, chromatin modification|GO:0016569, covalent chromatin modification|GO:0009892, negative regulation of metabolic process|GO:0009890, negative regulation of biosynthetic process|GO:0018022, peptidyl-lysine methylation|GO:0010629, negative regulation of gene expression|GO:0006807, nitrogen compound metabolic process|GO:0050789, regulation of biological process|GO:0097659, nucleic acid-templated transcription|GO:1901576, organic substance biosynthetic process|GO:0044260, cellular macromolecule metabolic process|GO:0016043, cellular component organization|GO:0065007, biological regulation|GO:0071840, cellular component organization or biogenesis|GO:0032259, methylation|GO:0018130, heterocycle biosynthetic process|GO:0009889, regulation of biosynthetic process|GO:0050794, regulation of cellular process|GO:0043412, macromolecule modification|GO:0036211, protein modification process|GO:0043414, macromolecule methylation|GO:0008152, metabolic process|GO:0034654, nucleobase-containing compound biosynthetic process|GO:0016070, RNA metabolic process|GO:0044271, cellular nitrogen compound biosynthetic process|GO:0006355, regulation of transcription, DNA-templated|GO:0010556, regulation of macromolecule biosynthetic process|GO:0006351, transcription, DNA-templated|GO:0010558, negative regulation of macromolecule biosynthetic process|GO:0032774, RNA biosynthetic process|GO:0044249, cellular biosynthetic process|GO:0034641, cellular nitrogen compound metabolic process|GO:0034645, cellular macromolecule biosynthetic process|GO:0044699, single-organism process|GO:0006139, nucleobase-containing compound metabolic process|GO:0043933, macromolecular complex subunit organization|GO:0034968, histone lysine methylation|GO:0009987, cellular process|GO:0006725, cellular aromatic compound metabolic process|GO:1903506, regulation of nucleic acid-templated transcription|GO:1903507, negative regulation of nucleic acid-templated transcription|GO:0045892, negative regulation of transcription, DNA-templated|GO:0048519, negative regulation of biological process|GO:0008213, protein alkylation|GO:0051253, negative regulation of RNA metabolic process|GO:0051252, regulation of RNA metabolic process|GO:0043170, macromolecule metabolic process|GO:0031327, negative regulation of cellular biosynthetic process|GO:0031326, regulation of cellular biosynthetic process|GO:0031324, negative regulation of cellular metabolic process|GO:0031323, regulation of cellular metabolic process|GO:0090304, nucleic acid metabolic process|GO:0006325, chromatin organization|GO:2000112, regulation of cellular macromolecule biosynthetic process|GO:2000113, negative regulation of cellular macromolecule biosynthetic process|GO:0071704, organic substance metabolic process|GO:0010467, gene expression|GO:0010468, regulation of gene expression|GO:0045934, negative regulation of nucleobase-containing compound metabolic process|GO:0044267, cellular protein metabolic process|GO:0019219, regulation of nucleobase-containing compound metabolic process|GO:0006464, cellular protein modification process|GO:1902679, negative regulation of RNA biosynthetic process|GO:0009058, biosynthetic process|GO:0009059, macromolecule biosynthetic process|GO:0044763, single-organism cellular process|GO:0051171, regulation of nitrogen compound metabolic process|GO:0051172, negative regulation of nitrogen compound metabolic process|GO:0006996, organelle organization|GO:0044238, primary metabolic process|GO:0051276, chromosome organization|GO:0044237, cellular metabolic process|GO:1902589, single-organism organelle organization|GO:0048523, negative regulation of cellular process|GO:1901564, organonitrogen compound metabolic process	GO:0043231, intracellular membrane-bounded organelle|GO:0005634, nucleus|GO:0044464, cell part|GO:0005623, cell|GO:0005622, intracellular|GO:0043229, intracellular organelle|GO:0044424, intracellular part|GO:0043227, membrane-bounded organelle|GO:0043226, organelle	GO:0018024, histone-lysine N-methyltransferase activity|GO:0043167, ion binding|GO:0042054, histone methyltransferase activity|GO:0046914, transition metal ion binding|GO:0003824, catalytic activity|GO:0008757, S-adenosylmethionine-dependent methyltransferase activity|GO:0043169, cation binding|GO:0016740, transferase activity|GO:0016278, lysine N-methyltransferase activity|GO:0016279, protein-lysine N-methyltransferase activity|GO:0016741, transferase activity, transferring one-carbon groups|GO:0008170, N-methyltransferase activity|GO:0008276, protein methyltransferase activity|GO:0008270, zinc ion binding|GO:0046976, histone methyltransferase activity (H3-K27 specific)|GO:0008168, methyltransferase activity|GO:0046872, metal ion binding|GO:0042800, histone methyltransferase activity (H3-K4 specific)|GO:0005488, binding		http://www.uniprot.org/uniprot/G1LXP2	FALSE
29	G1MEC8	G1MEC8	NPAT	− 2.308730386	GO:0080090, regulation of primary metabolic process|GO:0019222, regulation of metabolic process|GO:0000082, G1/S transition of mitotic cell cycle|GO:0000083, regulation of transcription involved in G1/S transition of mitotic cell cycle|GO:1901362, organic cyclic compound biosynthetic process|GO:1901360, organic cyclic compound metabolic process|GO:0010605, negative regulation of macromolecule metabolic process|GO:0010604, positive regulation of macromolecule metabolic process|GO:0048518, positive regulation of biological process|GO:0048519, negative regulation of biological process|GO:0060255, regulation of macromolecule metabolic process|GO:2001141, regulation of RNA biosynthetic process|GO:0046483, heterocycle metabolic process|GO:0019438, aromatic compound biosynthetic process|GO:0009892, negative regulation of metabolic process|GO:0009893, positive regulation of metabolic process|GO:0009890, negative regulation of biosynthetic process|GO:0009891, positive regulation of biosynthetic process|GO:0010628, positive regulation of gene expression|GO:0050789, regulation of biological process|GO:0097659, nucleic acid-templated transcription|GO:0045934, negative regulation of nucleobase-containing compound metabolic process|GO:1901576, organic substance biosynthetic process|GO:0044260, cellular macromolecule metabolic process|GO:0065007, biological regulation|GO:0044699, single-organism process|GO:0006366, transcription from RNA polymerase II promoter|GO:0018130, heterocycle biosynthetic process|GO:0009889, regulation of biosynthetic process|GO:0050794, regulation of cellular process|GO:0008152, metabolic process|GO:0034654, nucleobase-containing compound biosynthetic process|GO:0016070, RNA metabolic process|GO:0044271, cellular nitrogen compound biosynthetic process|GO:0006355, regulation of transcription, DNA-templated|GO:0010557, positive regulation of macromolecule biosynthetic process|GO:0010556, regulation of macromolecule biosynthetic process|GO:0006351, transcription, DNA-templated|GO:0010558, negative regulation of macromolecule biosynthetic process|GO:0032774, RNA biosynthetic process|GO:0044249, cellular biosynthetic process|GO:0034641, cellular nitrogen compound metabolic process|GO:0034645, cellular macromolecule biosynthetic process|GO:0007049, cell cycle|GO:0006139, nucleobase-containing compound metabolic process|GO:1903508, positive regulation of nucleic acid-templated transcription|GO:0009987, cellular process|GO:0006725, cellular aromatic compound metabolic process|GO:1903506, regulation of nucleic acid-templated transcription|GO:1903507, negative regulation of nucleic acid-templated transcription|GO:0045893, positive regulation of transcription, DNA-templated|GO:0090304, nucleic acid metabolic process|GO:0051253, negative regulation of RNA metabolic process|GO:0051252, regulation of RNA metabolic process|GO:0051254, positive regulation of RNA metabolic process|GO:0043170, macromolecule metabolic process|GO:1902680, positive regulation of RNA biosynthetic process|GO:0006807, nitrogen compound metabolic process|GO:0031328, positive regulation of cellular biosynthetic process|GO:0031327, negative regulation of cellular biosynthetic process|GO:0031326, regulation of cellular biosynthetic process|GO:0031325, positive regulation of cellular metabolic process|GO:0031324, negative regulation of cellular metabolic process|GO:0031323, regulation of cellular metabolic process|GO:1903047, mitotic cell cycle process|GO:0044770, cell cycle phase transition|GO:0044772, mitotic cell cycle phase transition|GO:0022402, cell cycle process|GO:2000112, regulation of cellular macromolecule biosynthetic process|GO:0071704, organic substance metabolic process|GO:0010467, gene expression|GO:0006357, regulation of transcription from RNA polymerase II promoter|GO:0010468, regulation of gene expression|GO:0045935, positive regulation of nucleobase-containing compound metabolic process|GO:0000278, mitotic cell cycle|GO:0019219, regulation of nucleobase-containing compound metabolic process|GO:1902679, negative regulation of RNA biosynthetic process|GO:0009058, biosynthetic process|GO:0009059, macromolecule biosynthetic process|GO:0044763, single-organism cellular process|GO:0051171, regulation of nitrogen compound metabolic process|GO:0051172, negative regulation of nitrogen compound metabolic process|GO:0051173, positive regulation of nitrogen compound metabolic process|GO:0044843, cell cycle G1/S phase transition|GO:0044238, primary metabolic process|GO:0044237, cellular metabolic process|GO:0048523, negative regulation of cellular process|GO:0048522, positive regulation of cellular process	GO:0031974, membrane-enclosed lumen|GO:0043229, intracellular organelle|GO:0043227, membrane-bounded organelle|GO:0043226, organelle|GO:0005737, cytoplasm|GO:0016604, nuclear body|GO:0031981, nuclear lumen|GO:0005634, nucleus|GO:0005654, nucleoplasm|GO:0044451, nucleoplasm part|GO:0043231, intracellular membrane-bounded organelle|GO:0043233, organelle lumen|GO:0097504, Gemini of coiled bodies|GO:0044464, cell part|GO:0005623, cell|GO:0005622, intracellular|GO:0044446, intracellular organelle part|GO:0070013, intracellular organelle lumen|GO:0044428, nuclear part|GO:0044424, intracellular part|GO:0015030, Cajal body|GO:0044422, organelle part	GO:0008022, protein C-terminus binding|GO:0000989, transcription factor activity, transcription factor binding|GO:0047485, protein N-terminus binding|GO:0003714, transcription corepressor activity|GO:0003713, transcription coactivator activity|GO:0003712, transcription cofactor activity|GO:0005488, binding|GO:0005515, protein binding|GO:0000988, transcription factor activity, protein binding		http://www.uniprot.org/uniprot/G1MEC8	FALSE

### Functional annotation of differentially modulated proteins

**Figure 2:**
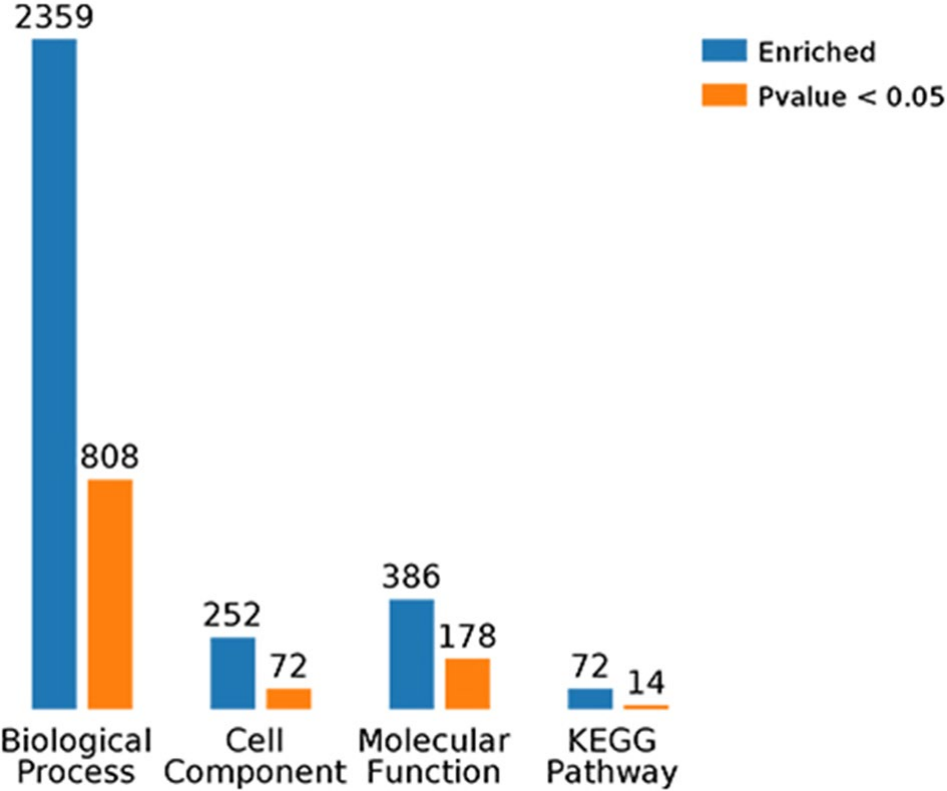
Number of enriched GO biological process, cell component and molecular function classifications and KEGG pathways defined by the differentially regulated proteins in the A vs B + C comparison. The bar chart shows the number of enriched categories with no statistical cut-off (blue) and with a threshold of p < 0.05.

**Figure 3:**
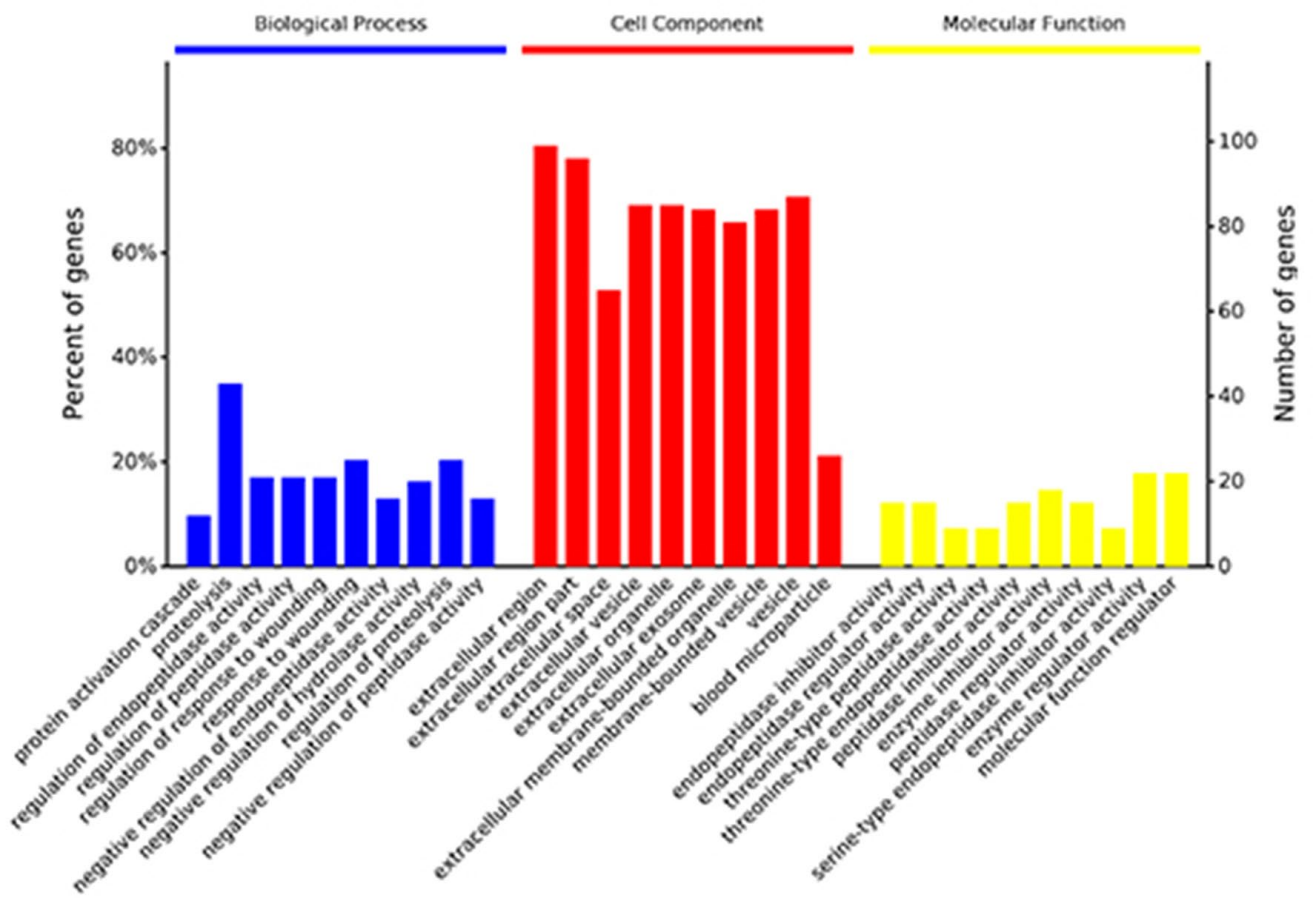
GO enrichment analysis showing the top-10 biological processes, cell compartments and molecular functions enriched among the proteins that differ in abundance between pandas with cataracts (band A) and those without cataracts (bands B + C). The enriched categories are arranged left to right by p-value (more significant on the left) and the bars represent the percentage of all differentially regulated proteins included in the category.

**Figure 4:**
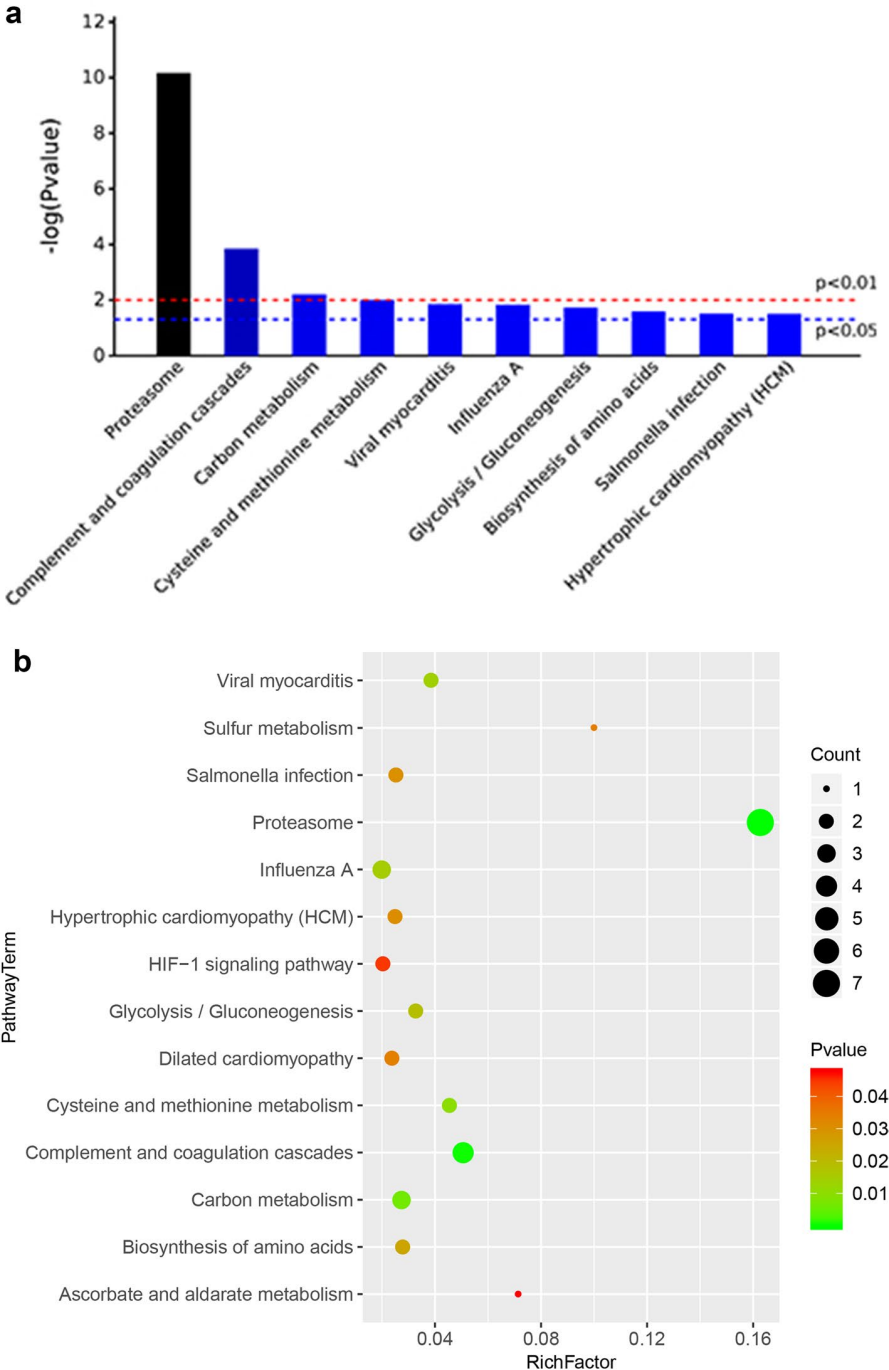
KEGG pathway enrichment analysis. (**a**) The top-10 pathways enriched among the proteins that differ in abundance between pandas with cataracts (band A) and those without cataracts (bands B + C). The enriched pathways are arranged left to right by p-value (more significant on the left) which is also represented by the logarithmic scale of the y-axis. The dotted lines represent cut-offs at significance values of p < 0.05 and p < 0.01. (**b**) Significant enrichment functional scatter plot in which the y-axis represents functional annotation, and the x-axis represents the Rich factor of that function (the number of differential genes enriched in a pathway divided by the total number of genes annotated in that pathway). The p-value is represented by the color of the dots, and the number of differentially expressed genes representing each function is shown by the size of the dots. Only the 14 highest enrichments are shown.

The biological functions and related pathways of the 148 differentially modulated proteins (A vs B + C) were investigated by screening the sequences against the GO and KEGG databases (Fig. 2). This revealed a total of 2359 enriched biological process classifications as well as 252 cell components, 386 molecular functions and 72 KEGG pathways. When the hits were restricted by a significance of p < 0.05, the number of enriched biological processes fell to 808, as well as 72 cell components, 178 molecular functions and 14 KEGG pathways. The top 10 enriched biological processes, cell components and molecular functions based on significance are shown in Fig. 3. Interestingly, most of the top-ranking functions related to proteolysis and its regulation, which is a significant factor in the age-related deterioration of mammalian tissues and would be expected in the context of cataract formation. The top-ranking molecular functions were in broad agreement, with most of them directly involved in the regulation of peptidases or classified more generally as regulators of enzyme activity. Remarkably, the most significant cell compartment classifications indicated extracellular activity and represented more than half of all the proteins, suggesting that cataract formation in pandas is strongly associated with the extracellular regulation of protein turnover. The inclusion of wounding responses among the biological functions may indicate the response to oxidative stress associated with aging and the formation of cataracts. In line with these results, the analysis of enriched KEGG pathways revealed the proteasome in the top-ranking position by a long margin (Fig. 4a) and also the pathway with the most significant Rich factor (Fig. 4b). The proteasome is the key regulatory complex responsible for protein turnover and its gradually declining activity is known to be associated with aging, thus providing a potential explanation for its link with the formation of cataracts.

### Protein interaction analysis

**Figure 5:**
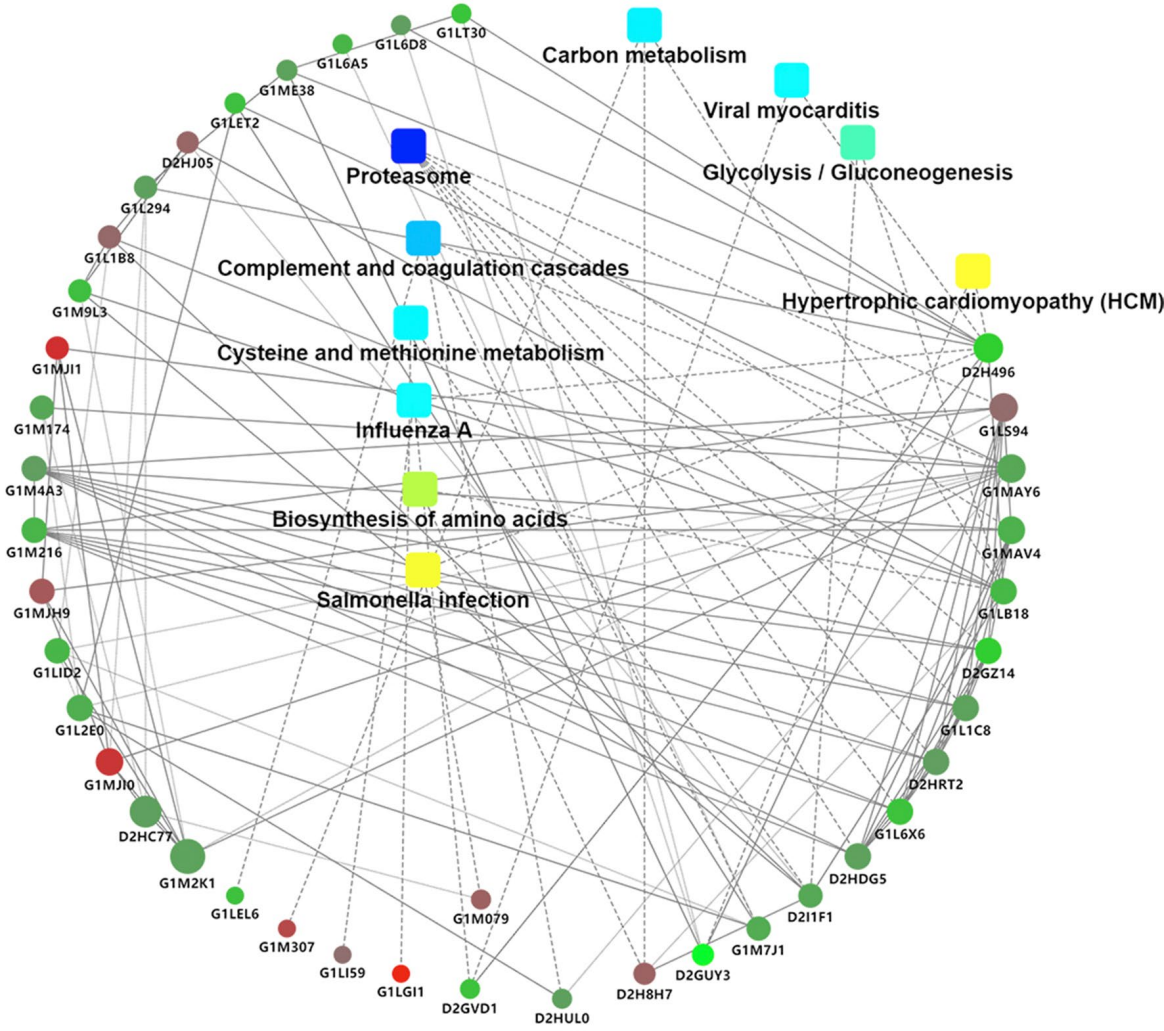
Protein interaction network visualized using the Omicsbean platform. Circles around the edge of the diagram represent the differentially regulated proteins (red = upregulated, green = downregulated, with different shades representing the fold change). The squares represent biological processes, cell localization, molecular functions or signaling pathways, and the color code indicates significance (yellow = low, blue = high, with deeper shades indicating greater significance). Solid lines represent protein–protein interactions, and dotted lines represent the involvement of proteins in metabolic/molecular pathways or functions.

Interactions between the differentially expressed proteins and KEGG pathways were visualized by integrating the data using the online platform Omicsbean to generate the network diagrams shown in Fig. 5. As well as showing that many of the differentially modulated proteins interact directly with each other, the diagrams revealed that the downregulated proteins were responsible for most of the predicted interactions and were almost exclusively responsible for interactions with the proteasome. This suggests that one of the key events during age-related cataract formation in pandas is the depletion or loss of proteasome components, which would contribute to the inability of the proteasome to maintain protein integrity in response to oxidative stress.

## Discussion

Aging humans and other mammals can develop cataracts due to the accumulation of oxidative damage in the lens, and this is associated with a declining quality of life^1^. In captive giant pandas, which can live 10–15 years longer than animals in the wild^3,4^, the prevalence of cataracts in the aged population (≥ 19 years old) has now reached ~ 20%. The development of age-related cataracts is mainly promoted by environmental factors that cause oxidative damage to DNA and proteins^5–7,9^. This triggers changes in DNA methylation and gene expression, and in previous studies we and others have identified target genes involved in apoptosis, DNA repair and oxidative stress responses that may represent an attempt to counter or reverse the damage that occurs during cataract formation^17,18,24–26^.

Epigenetic modifications such as DNA methylation can directly affect transcription, and we have previously reported correlations between the methylated loci detected in panda cataracts and the modulation of gene expression in affected vs unaffected individuals^17,18^. However, the regulation of gene expression at the level of protein synthesis and turnover means that transcript and protein levels often do not correlate directly, and this is particularly relevant in the lens where the proteome is known to have a very slow turnover compared to non-lens tissues in the eye, suggesting that serum markers of cataract formation may not track closely with mRNA levels^27^. We therefore used TMT quantitative proteomics to compare serum from affected and unaffected pandas in order to directly investigate the proteins that change in abundance during cataract formation.

The comparison of affected females (four specimens) with unaffected males and females as a single group (six specimens) revealed 148 differentially modulated proteins, 69 upregulated and 79 downregulated in the pandas with cataracts. Functional annotation of these proteins revealed a broad range of associations, but the most significant were related to the biological processes of proteolysis and its regulation, and molecular functions involving the regulation of peptidases and other enzymes. This was supported by KEGG pathway enrichment analysis, which linked many of the differentially expressed proteins to the proteasome, a protein complex that removes damaged and otherwise surplus proteins by proteolysis^28^. This link was further supported by protein interaction network analysis, which clearly showed the proteasome as a core interaction hub for the differentially expressed proteins, especially those that were downregulated in the affected pandas. A survey of the list of differentially expressed proteins revealed nine known proteasome subunits, eight of which were downregulated (PSMA7, PSMB1, PSMB7, PSMB4, PSMA6, PSMA4, PSMA3 and PSMB8) while only one was upregulated (PSMA2). The list also included a large number of peptidase inhibitors, most of which were downregulated (e.g., APOA2, SERPINC1, SERPING1, ITH1, ITH2, KNG1, FETUB, SERPINA1 and AHSG) which also indicates that cataract formation involves the dysregulation of the protein turnover apparatus. Abnormalities affecting the proteasome that allow the accumulation of protein aggregates have been linked to human cataracts, including genetic variants of PSMC3^29^, but the overexpression of the proteasome activator PA28αβ did not rescue induced cataracts in mice^30^. This suggests the proteasome is already highly active in lens tissue and its activity cannot be enhanced using activators, but the loss of activity increases the likelihood of cataracts, as previously reported^31,32^.

One of the most striking results from the GO enrichment analysis was the overrepresentation of extracellular proteins, accounting for more than 50% of all the differentially regulated proteins we identified. It is well known that one of the key functions of the proteasome is to remove advanced glycation end-products (AGEs), which can form both inside and outside the cells, contributing to the morbidity associated with aging, including cataracts^33^. Such products also progressively inhibit the activity of the proteasome, leading to a positive feedback cycle of proteotoxic stress that is ultimately pathogenic^34^. The enrichment we observed for extracellular proteins may reflect the lens attempting to deal with the accumulation of extracellular AGEs during cataract formation. One well-known example of a lens protein that forms AGEs is αA-crystallin, although this accumulates inside cells^35^, but lens capsule proteins are also glycated upon aging, and thus contribute to cataract formation^36^. Several of the differentially regulated proteins we detected were annotated as proteins that bind RAGE receptors, including S100A12, S100AB, S100A9 and PSMA2, all of which were upregulated. However, we note that the preponderance of extracellular proteins in our differentially regulated dataset could have a more prosaic explanation—that extracellular proteins are more likely to enter the bloodstream than intracellular proteins are therefore more likely to be captured as serum markers. This may also explain the detection of proteins related to the coagulation pathway such as plasma kallikrein. However, several of the differentially regulated extracellular proteins we detected are components of the extracellular matrix that have previously been associated with cataract formation^37^. For example, collagen 1A2 and the integrin-binding protein vitronectin were both downregulated in the affected animals (along with the intracellular integrin-binding protein TLN1), supporting the reported role of the extracellular matrix and integrin signaling in lens development and cataract formation^38,39^. Interestingly multiple keratin proteins were upregulated in the animals with cataracts (KRT18, KRT75, KRT1 and KRT10) whereas various cytoskeletal components and their interaction partners were downregulated, suggesting a complex network of signaling inside and outside the cell as a stress response to the accumulation of protein aggregates.

Although the above results paint an intriguing picture of the panda proteome related to cataract formation, it is important to highlight some weaknesses of the study that may influence the results. First, we acknowledge that the sample number is small, which reflects the limited availability of samples and the further limitations imposed by the ethical committee. This has the potential to introduce bias into the results causes by undetected disease in the test subjects despite our careful and strict inclusion criteria. Second, almost all recorded instances of captive giant pandas with age-related cataracts are female—only two male cases have been reported, one deceased with no material available and one living specimen. This has the potential to introduce bias into the results caused by sex-dependent factors, although we addressed this to a certain extent by dividing the control group into sex categories to enable female affected vs female unaffected and male unaffected vs female unaffected comparisons. These comparisons revealed some differences between males and females but not in the same pathways that discriminated between affected and unaffected animals, suggesting there is negligible sex-dependent interference.

In conclusion, we have generated multiple lines of evidence based on quantitative proteomics showing that age-related cataracts in pandas involve the dysregulation of the lens proteome resulting in the detection of nearly 150 positive and negative protein markers in the blood. More than half of these markers are extracellular proteins, suggesting either that cataract formation causes the extensive modification of the extracellular proteome or that extracellular proteins are more likely to enter the bloodstream in sufficient quantities for detection. One of the most prominent aspects of the cataract serum profile was the depletion of proteasome components and their interaction partners, supporting previous results showing that the proteasome maintenance system deteriorates with age and is progressively less responsive to the accumulation of protein aggregates. Such aggregates may then exacerbate the problem by directly inhibiting the proteasome. Interestingly, we did not identify a single transcription factor among the differentially regulated proteins we detected, which indicates that the response to cataract formation is largely post-transcriptional. The identification of multiple protein markers correlating with the presence of cataracts could allow the development of bioassays for the early detection of cataracts in captive animals, based either on the most profound changes in abundance of key proteins such as those listed in Table 3, or the analysis of multiple biomarker profiles to define cataract-positive patterns, as shown for other diseases^40,41^. In terms of clinical applications, proteins that increase in abundance during the formation of cataracts could be tested as new drug targets, whereas those depleted during the formation of in cataracts could be evaluated as candidates for replacement therapy, leading to new opportunities for the prevention and/or treatment of cataracts in aging captive pandas.

### Supplementary Information

Supplementary Legends.

Supplementary Figure S1.

Supplementary Figure S1.

Supplementary Figure S2.

Supplementary Figure S2.

Supplementary Figure S3.

Supplementary Table S1.

## Data Availability

The datasets generated during the current study are available in the ProteomeXchange Consortium repository, http://proteomecentral.proteomexchange.org/cgi/GetDataset?ID=PXD031039 or https://www.iprox.cn/page/project.html?id=IPX0004000000, Project ID IPX0004000000, accession number PXD031039.
