# Supplementary material for: Comparative proteomics in captive giant pandas to identify proteins involved in age-related cataract formation
Source: Sci Rep. 2023 Aug 5;13:12722. doi: 10.1038/s41598-023-40003-0 (PMC10404263; doi:10.1038/s41598-023-40003-0)
Supplement: Supplementary file 1 — Supplementary Legends. [file 41598_2023_40003_MOESM1_ESM.docx]

**Supplementary Figure S1**. Differential protein expression when comparing band A (female pandas with cataracts) to band B (female pandas without cataracts). **(a)** The number of upregulated and downregulated proteins in band A compared to band B. **(b)** Volcano plot showing the most meaningful differentially expressed proteins in band A compared to band B by plotting significance on the y-axis against fold-change on the x-axis. Proteins to the left (green dots) are downregulated and those to the right (red dots) are upregulated according to the statistical threshold, below which the proteins are not considered to be differentially expressed (black dots).

**Supplementary Figure S2**. Differential protein expression when comparing band A (female pandas with cataracts) to band C (male pandas without cataracts). **(a)** The number of upregulated and downregulated proteins in band A compared to band C. **(b)** Volcano plot showing the most meaningful differentially expressed proteins in band A compared to band C by plotting significance on the y-axis against fold-change on the x-axis. Proteins to the left (green dots) are downregulated and those to the right (red dots) are upregulated according to the statistical threshold, below which the proteins are not considered to be differentially expressed (black dots).

**Supplementary Figure S3**. Heat map showing the expression levels of the identified differentially regulated proteins (y-axis) in each specimen (x-axis). Each row represents the amount of each protein expressed in each specimen, with darker colors indicating greater abundance, and each column represents the amount of all the different proteins expressed in each group. The top tree shows the results of clustering analysis for different groups of protein data, and the left tree shows the results of clustering analysis for different groups of protein data.
