# Supplementary figures and images for: Comparative proteomics in captive giant pandas to identify proteins involved in age-related cataract formation
Source: Sci Rep. 2023 Aug 5;13:12722. doi: 10.1038/s41598-023-40003-0 (PMC10404263; doi:10.1038/s41598-023-40003-0)

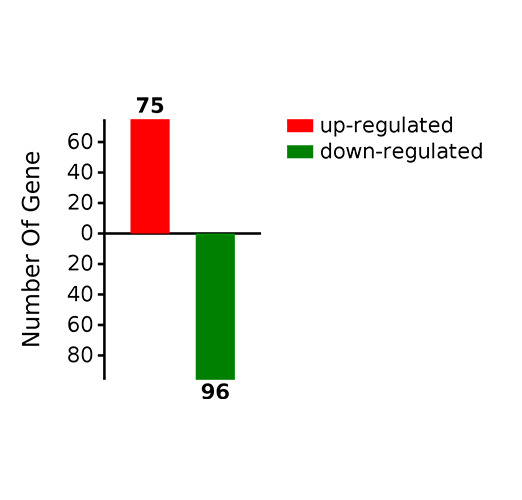

Supplement: Supplementary file 2 — Supplementary Figure S1. [file 41598_2023_40003_MOESM2_ESM.jpg]

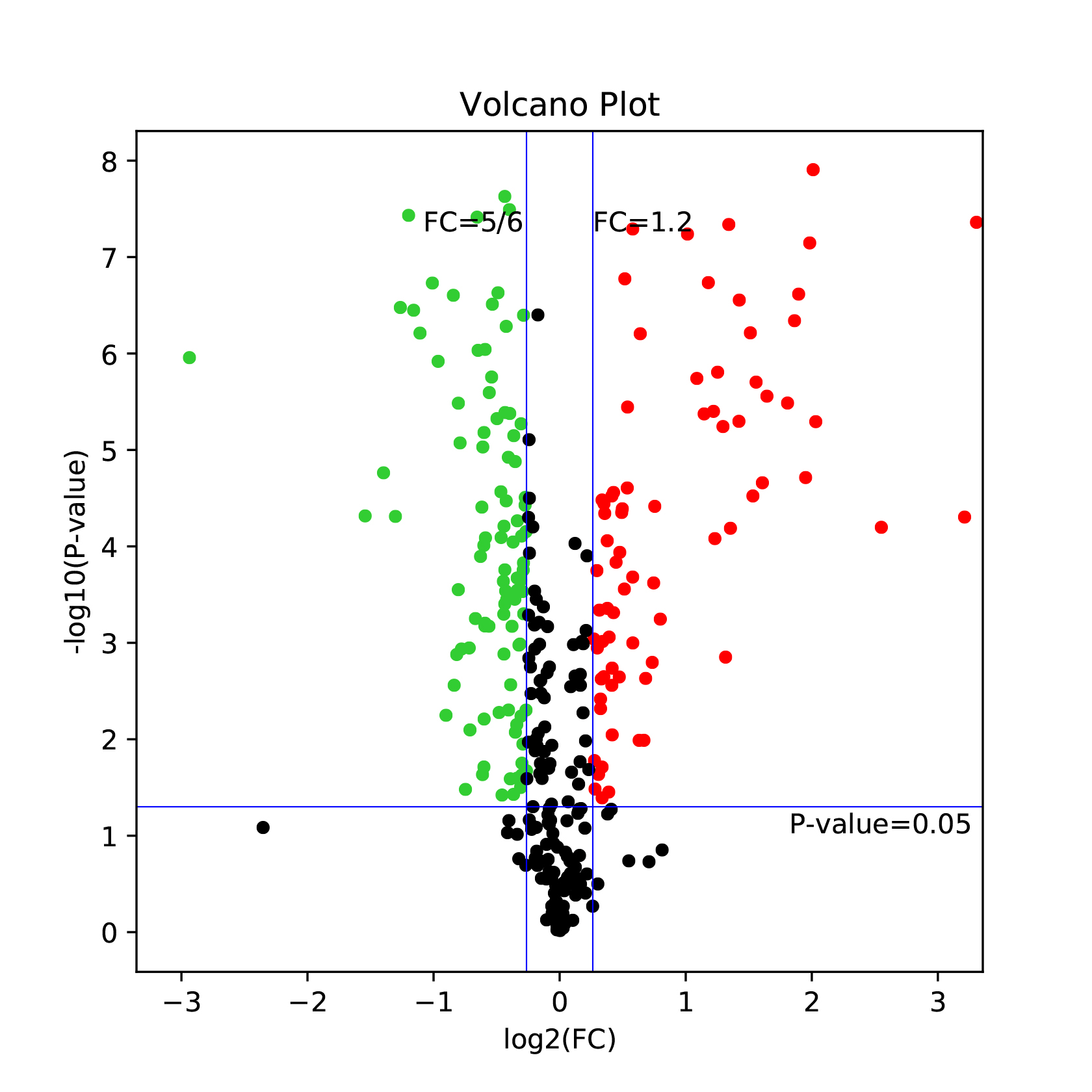

Supplement: Supplementary file 3 — Supplementary Figure S1. [file 41598_2023_40003_MOESM3_ESM.jpg]

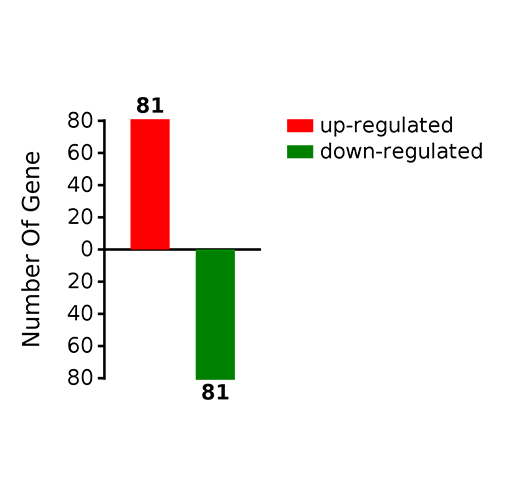

Supplement: Supplementary file 4 — Supplementary Figure S2. [file 41598_2023_40003_MOESM4_ESM.jpg]

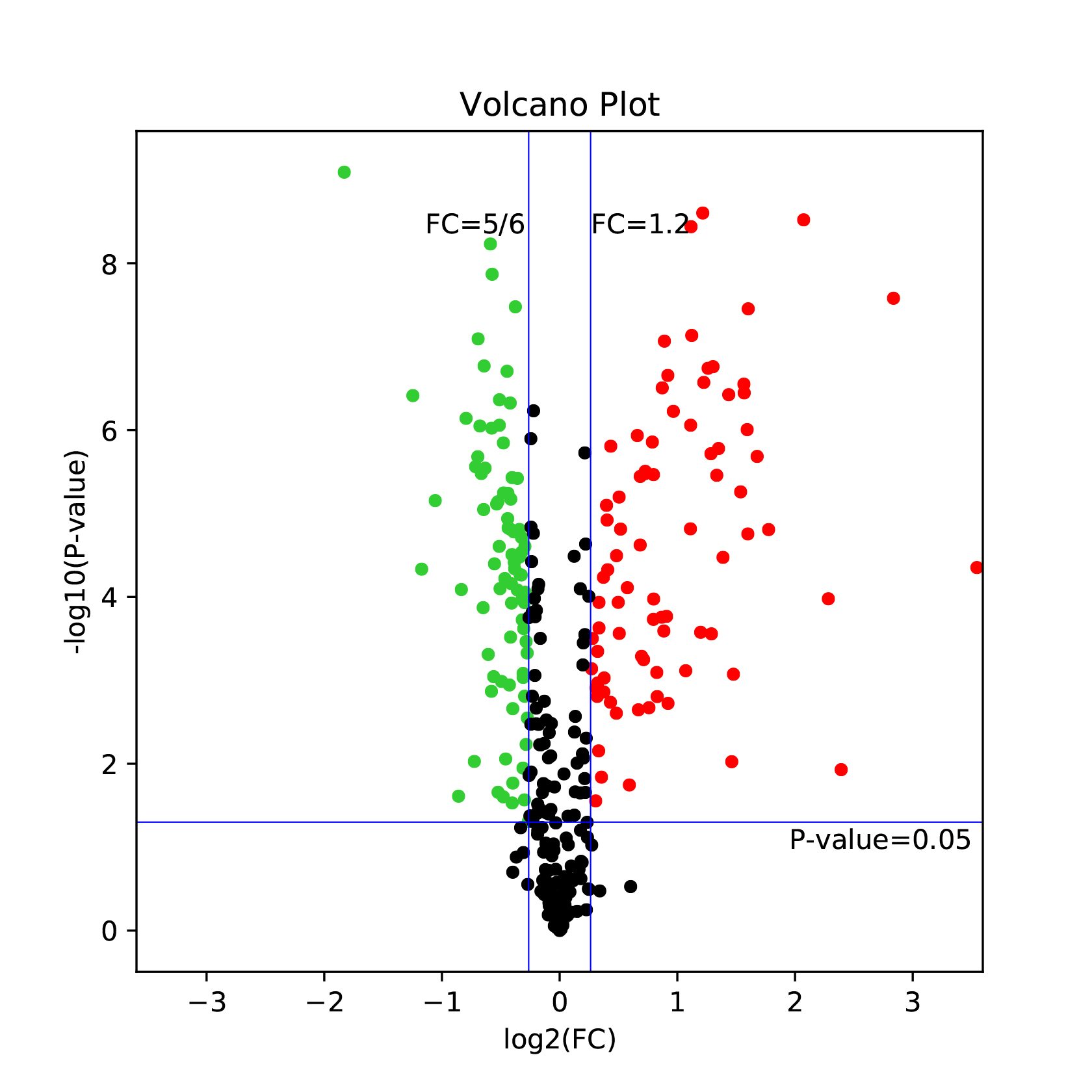

Supplement: Supplementary file 5 — Supplementary Figure S2. [file 41598_2023_40003_MOESM5_ESM.jpg]

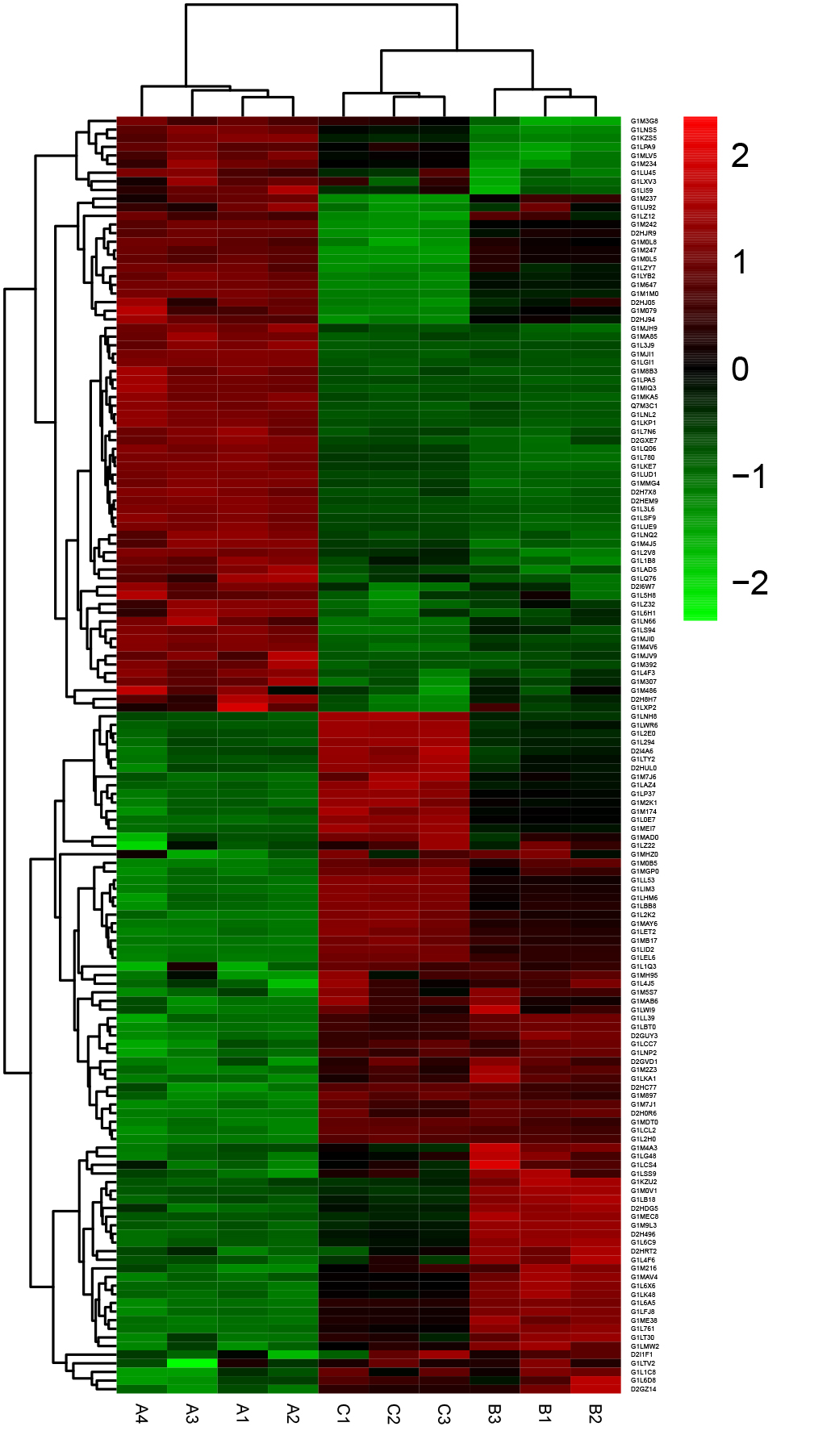

Supplement: Supplementary file 6 — Supplementary Figure S3. [file 41598_2023_40003_MOESM6_ESM.jpg]
